# Surface states of dual-atom catalysts should be considered for analysis of electrocatalytic activity

**DOI:** 10.1038/s42004-022-00810-4

**Published:** 2023-01-06

**Authors:** Weijie Yang, Zhenhe Jia, Binghui Zhou, Li Wei, Zhengyang Gao, Hao Li

**Affiliations:** 1 Department of Power Engineering, School of Energy, Power and Mechanical Engineering, North China Electric Power University, 071003 Baoding, China; 2 School of Chemical and Biomolecule Engineering, The University of Sydney, Darlington, 2006 NSW Australia; 3 Advanced Institute for Materials Research (WPI-AIMR), Tohoku University, Sendai, 980-8577 Japan

**Keywords:** Electrocatalysis, Electrocatalysis, Structural properties, Computational chemistry

## Abstract

Experimentally well-characterized dual-atom catalysts (DACs), where two adjacent metal atoms are stably anchored on carbon defects, have shown some clear advantages in electrocatalysis compared to conventional catalysts and emerging single-atom catalysts. However, most previous theoretical studies directly used a pristine dual-atom site to analyze the electrocatalytic activity of a DAC. Herein, by analyzing 8 homonuclear and 64 heteronuclear DACs structures with ab initio calculations, our derived surface Pourbaix diagrams show that the surface states of DACs generally differ from a pristine surface at electrocatalytic operating conditions. This phenomenon suggests that the surface state of a DAC should be considered before analyzing the catalytic activity in electrocatalysis, while the electrochemistry-driven pre-adsorbed molecules generated from the liquid phase may either change the electronic properties or even block the active site of DACs. Based on these results, we provide a critical comment to the catalyst community: before analyzing the electrocatalytic activity of a DAC, its surface state should be analyzed beforehand.

## Abstract (web summary)

Dual-atom catalysts (DACs) pose several advantages such as uniformity in the active sites and high atom utilization efficiency supported by a synergy of the two catalyst metal atoms. Here, the authors use spin-polarized density functional theory with van der Waals corrections to derive surface Pourbaix diagrams of an Fe–Ni–N_x_–C model, showing that the surface states of DACs generally differ from a pristine surface at electrocatalytic operating conditions due to the strong adsorption capacity of a DAC’s unique metal–metal bridge site.

## Introduction

Electrocatalysis furnishes one credible alternative for the conversion of sustainable energy. Most electrocatalytic reactions generally occur at the electrode–liquid interface and involve proton and electron transfer^1^. While the water dissociation reactions may occur following the steps:

1 $${{{{{{\rm{H}}}}}}}_{2}{{{{{\rm{O}}}}}}+* \leftrightarrow {{{{{{\rm{HO}}}}}}}^{* }+{{{{{{\rm{e}}}}}}}^{-}+{{{{{{\rm{H}}}}}}}^{+}$$

2 $${{{{{{\rm{H}}}}}}}_{2}{{{{{\rm{O}}}}}}+* \leftrightarrow {{{{{{\rm{O}}}}}}}^{* }+{2{{{{{\rm{e}}}}}}}^{-}+{2{{{{{\rm{H}}}}}}}^{+}$$

(∗ denotes a surface site), the catalyst surface active site may be occupied by additional cations/anions originated from the liquid dissolvent, resulting in a dynamic equilibrium state that is distinctive to its pristine form^2^. Therefore, probing the actual catalyst surface is particularly important before analyzing its electrocatalytic activity.

In electrocatalysis, knowing the state of a catalyst surface is essential because it determines the availability of surface-active sites, while the activity of a site is sensitive to many factors such as the coordination environment due to the electronic (ligand) and ensemble (site geometric) effects^3^. However, how to study the surface state during electrocatalysis is a hard nut to crack due to the lack of precise in situ methods to probe the surface during electrochemistry. To address this challenge, the surface Pourbaix diagram, which reveals the surface state as the function of pH and electrochemical potential based upon ab initio calculations^4^, emerged as an essential analytical method to help understand the catalyst surface under electrochemical conditions in liquid. Nørskov and colleagues^5^ applied surface Pourbaix diagrams to analyze the electrochemically most favorable surface states for Pt, Ag, and Ni for oxygen reduction reaction (ORR). They found that the theoretical ORR activity of the Ni surface changes dramatically if the adsorbate coverage is considered. Vinogradova et al.^6^ subsequently analyzed the surface Pourbaix diagrams of several transition metals and found that the Ir, Rh, and Ru surfaces will be significantly oxidized to a surface covered by ~1/3 monolayer HO* at ORR conditions. However, surface state analysis has been dismissed in many electrocatalytic studies until recent years when several studies have demonstrated the remarkable significance of analyzing the state of a catalyst surface under operating conditions, especially for transition metal oxides^7–9^. All these cases suggest that it is the specific surface state at operating conditions that predominate the activity of an electrocatalyst. So far, most of the reported surface state analyses mainly focused on metal single-crystal and transition metal oxide surfaces.

**Fig. 1:**
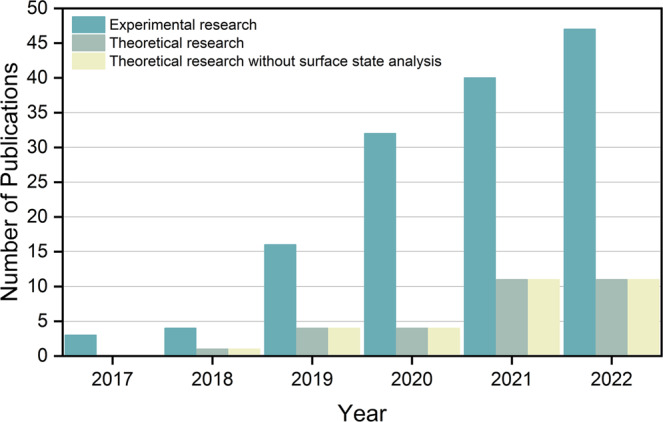
Statistics of DAC research. The number of experimental and theoretical reports on dual-atom electrocatalysts during the past 6 years. Data summarized from Web of Science.

Single-atom catalysts (SACs) have shown tremendous potentials in improving the catalytic activity and selectivity of electrocatalysis in part due to their tunable coordination environments and unique electronic structures^10,11^. However, while most of the SAC-related studies assumed a bare metal site as the active center for catalysis, very few previous studies analyzed the state of a SAC active center. Dobrota and co-workers^12^ analyzed the surface Pourbaix diagrams of SACs with metal atoms embedded into the N_*4*_-C moiety (where N and C represent nitrogen and carbon, respectively) of a graphene substrate. They displayed how the adsorption of H*, O*, and HO* could cause a blockage of active sites and alter the electronic structure of SACs. In most cases of SACs, the metal active centers are not poisoned by oxygen-containing adsorbates at a moderate potential, such as Ni-N_*4*_-C, where the potential is −1.4 to 1.1 V at pH = 0, and the surface remains pristine^12^. Nevertheless, SACs usually have only one active center, inhibiting the multiatomic pathways, e.g., the reaction that follows a Langmuir–Hinshelwood mechanism^13^. In contrast, the emerging dual-atom catalysts (DACs), since the pioneering work of the successful fabrication and characterization of graphene-supported Fe dopant pairs by He et al.^14^, provide more possible functions for electrocatalysis. Very recently, DACs have become an emerging topic in the catalysis community—many related research works have been reported with convenient synthetic routes and clear characterizations^15,16^. An increasing number of homonuclear and heteronuclear DACs are used in both experimental and theoretical research (Fig. 1)^17–19^.

Different from SACs, which mostly adsorb O* at the metal-atop site with weak O-metal bonding due to a strong repulsion^20^, DACs can provide metal-metal bridge sites that may bind O* much more strongly due to a higher coordination number of O*^21^. However, the dynamic equilibrium state between the reaction surface and other molecules was mostly unknown: whether the actual surface state of DACs is identical to the pristine surface is also an essential but often-overlooked question. To the best of our knowledge (as summarized in Fig. 1), almost all previous theoretical research dismissed the analysis of a DAC surface state under electrochemical operating conditions.

Motivated by the current research status, herein, we employed spin-polarized density functional theory calculations with van der Waals corrections (DFT-D3) to derive the surface Pourbaix diagrams of DACs. We consider the interaction between solution and catalyst as well as the coordination environment of the metal dual-atoms. Based on the Gibbs adsorption free energies of H*, O*, and HO* on DACs, surface Pourbaix diagrams were developed to distinguish the site occupation of 8 homonuclear and 64 heteronuclear DACs. Fe and Ni were selected for analyzing heteronuclear DACs they are widely studied both experimentally and theoretically (M-M’-N_*x*_-C, where M = Fe, M’ = Ni, *x* represents the detailed N configuration)^22,23^. The surface Pourbaix diagrams show that the active center including the atop and bridge sites of most models will be occupied by H*, O*, and HO*, which will significantly affect the reaction activity. Furthermore, this research breaks the conventional concept that the pristine form is the initial surface of DACs in electrocatalytic reactions and proposes the urgent need to probe the actual surface state of DACs either from experiment or theory.

## Results

### Catalyst structures

**Fig. 2:**
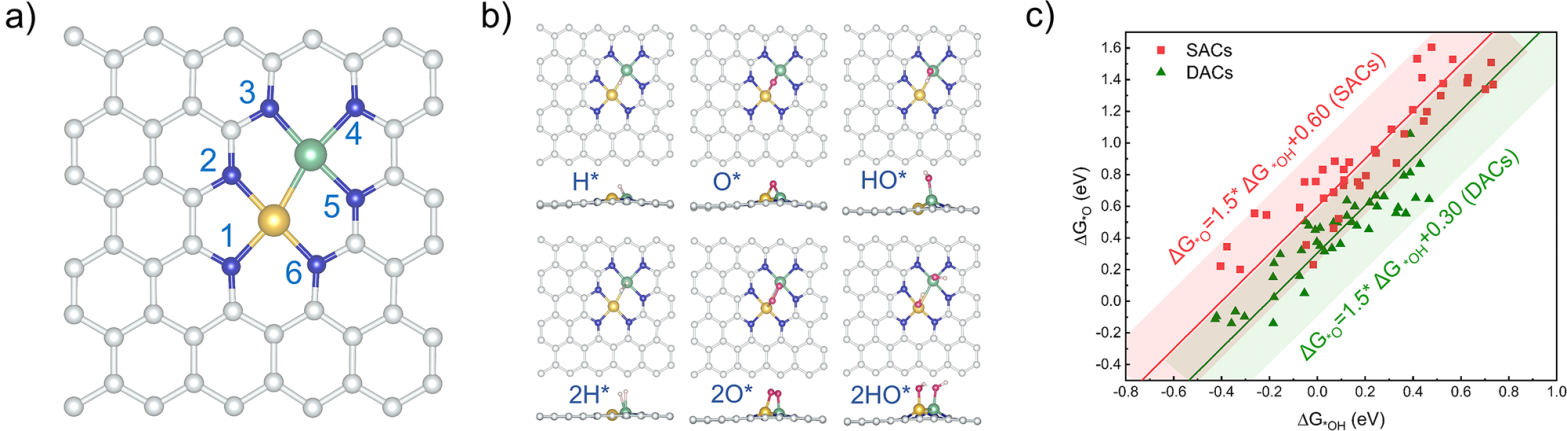
Structures and adsorption properties of DACs. **a** Structure and coordinating environment of Fe-Ni-N_*1,2,3,4,5,6*_-C, where yellow, green, blue, and silvery spheres represent Ni, Fe, N, and C, respectively. **b** Optimized configurations with the DAC adsorbed with 1H*, 2H*, 1O*, 2O*, 1HO*, and 2HO*, using Fe-Ni-N_*1,2,3,4,5,6*_-C as the example. Red and light pink spheres represent O and H, respectively. **c** Linear scaling relations of O^∗^ vs. HO* adsorption energies at SACs and DACs, where red and green data points represent SACs and DACs, respectively.

Stability analysis should always be the priority before the subsequent activity analysis^24^. In this work, we selected a Fe-Ni-N_*6*_-C model (Fig. 2a), a proven stable dual-atom structure^22,23^, as an example for illustration. We controlled the metal atom coordination environment by tuning the number of nitrogen atoms and location, affording a total of 64 DAC models. The nitrogen atoms were assigned as site-*x* (*x* = 1–6; the site collineating with the higher atomic number metal is named site-1). Clockwise along site-1, the other sites are in turn named: site-2, site-3, site-4, site-5, and site-6, respectively, to distinguish a configuration. As an example, the model in Fig. 2a is named Fe-Ni-N_*1,2,3,4,5,6*_-C. Because various coordination modes may co-exist in experimental synthesis^25^, we considered various coordination environments of DACs in our analysis. Besides, we consider that this type of DAC structure can be kinetically stable under proper synthetic conditions and a mild electrochemical environment, and the formation energies of such quad-atom vacancy sites were generally exothermic^26^.

Adsorption configurations and surface Pourbaix diagrams of these models were built to explore the surface state under electrochemical conditions. We found that many intermediates prefer to adsorb on the bridge site (Fig. 2b). Inspired by this phenomenon, linear scaling relations between the adsorption energies of O* and HO* were analyzed for both SACs and DACs (Fig. 2c). Interestingly, with the same binding strength to HO*, DACs adsorb O* generally more strongly (i.e., with more negative adsorption-free energies) than SACs, which is because O* more stably adsorbs at the metal-metal bridge-site with a higher coordination number (Fig. 2b), compared to the atop-site adsorption of O* at SACs. This suggests that DACs may be easily poisoned by O* under anodic conditions. Since pre-adsorbed atoms or molecules can significantly influence the electronic structure of a surface, this will affect the electrocatalytic activity. Therefore, to analyze the electrocatalytic reaction processes, the catalyst’s surface state must be explored beforehand. Dismissing this critical analytical step will likely mislead the conclusion.

### Adsorption of H*, O*, and HO* at pH = 0

**Fig. 3:**
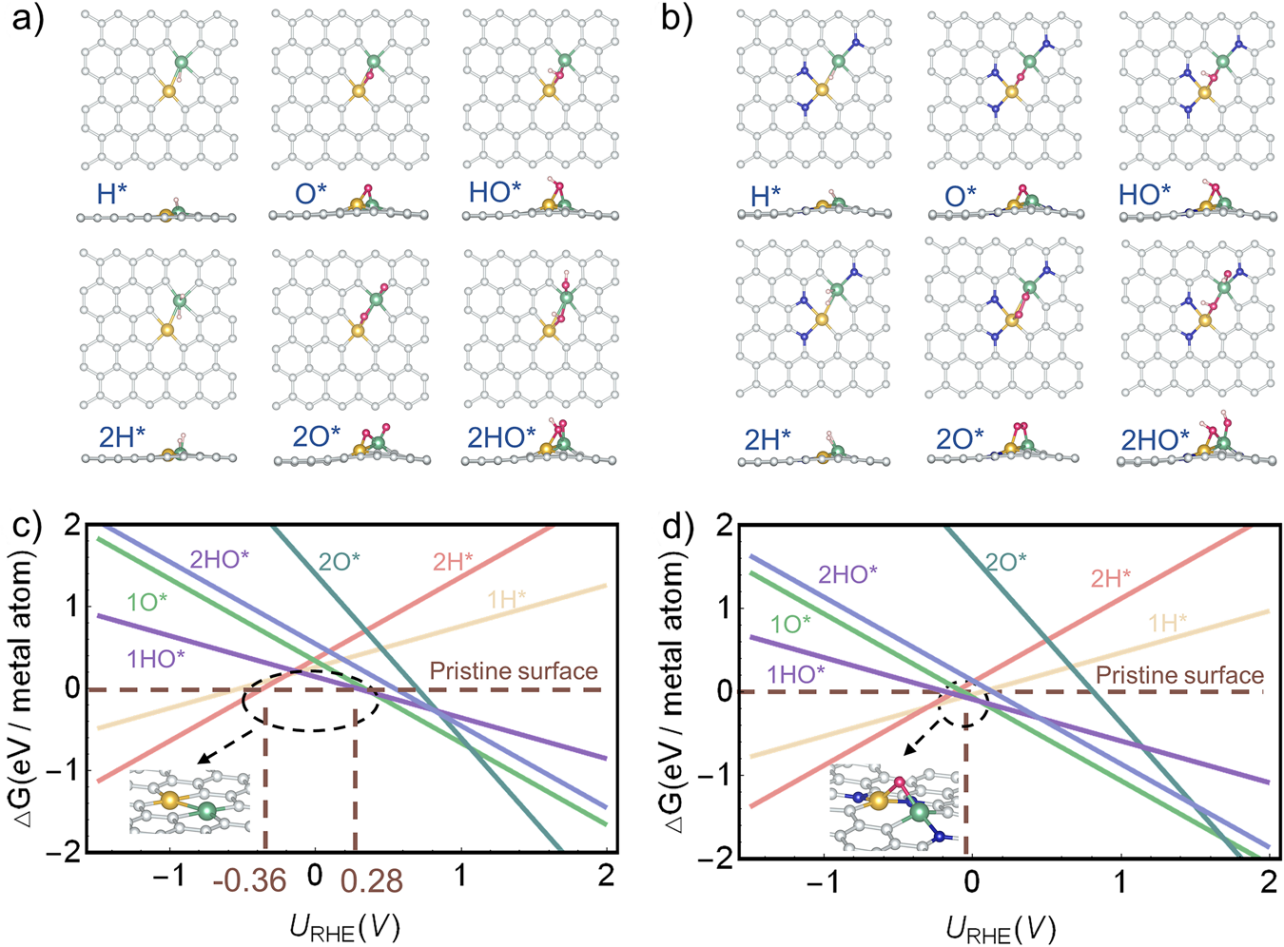
Examples of the surface Pourbaix diagrams of DACs at pH = 0. **a** Adsorption configurations of Fe-Ni-C and **b** Fe-Ni-N_*1,2,4*_-C, where yellow, green, blue, silvery, red, and light pink spheres represent Ni, Fe, N, C, O, and H, respectively. **c** Surface Pourbaix diagrams of Fe-Ni-C and **d** Fe-Ni-N_*1,2,4*_-C at pH = 0.

A surface Pourbaix diagram describes the relations between the potential and Gibbs free energy of DACs with various pre-adsorbed atoms or molecules generated from water. Herein, we developed the surface Pourbaix diagrams of 64 DAC models based on their adsorption configurations at pH = 0. Two representative catalysts, Fe-Ni-C (Fig. 3a, c) and Fe-Ni-N_*1,2,4*_-C (Fig. 3b, d) are used as examples to illustrate the analysis. Our calculations clearly show that the actual surface state varies at different potentials. Herein, we define a novel term, the electrochemical potential window (EPW), to describe the potential range in which a catalyst can retain its pristine state. As shown in Fig. 3c, Fe-Ni-C exhibits an EPW of –0.36 to 0.28 V, and its metal sites are pinned with H*, O*, or HO* outside this EPW. On the contrary, no such window is available for Fe-Ni-N_*1,2,4*_-C (Fig. 3d). Therefore, the surface Pourbaix diagrams suggest that the chemisorption of common electrocatalytic reactant molecules (e.g., O_2_, CO_2_, N_2_) is more likely within an available EPW. The varied surface Pourbaix diagrams obtained from our DAC models further suggest the metal site coordination environment can also alter the actual surface state. The above discussions further show that incorporating the catalyst surface state in the analysis under electrochemical operating conditions is crucial. Similar conclusions on other DACs are shown in Supplementary Tables 1 and 2.

According to the availability of the EPW, we can divide the 64 DACs into two categories: (1) the active sites are partially occupied (none, one, or two, e.g., Fe-Ni-C) within a certain potential range, and (2) the active sites are occupied at all potentials (e.g., Fe-Ni-N_*1,2,4*_-C). For a catalyst, if a reaction potential is within the EPW, then the pristine surface can be treated as the initial surface and suitable for the subsequent catalytic analysis. Otherwise, the actual surface state should be identified before further evaluation. For example, the pristine surface of the Fe-Ni-C, without any adsorbate at −0.35 V may be suitable for CO_2_ reduction reaction (CO_2_RR) because it is a typical CO_2_RR reaction potential (Fig. 3c). However, no pristine surface can be considered as the start for CO_2_RR over Fe-Ni-N_*1,2,4*_-C (Fig. 3d). Moreover, the surface of Fe-Ni-N_*1,2,4*_-C is occupied by the O* at 0 V (i.e., the typical potential of hydrogen evolution reaction, HER), so the subsequent calculations need to be started with the surface pre-adsorbed by an O* (Fig. 3d). In contrast, the corresponding initial surface of Fe-Ni-C for HER remains pristine. Therefore, probing the EPW is essential for understanding electrochemical reactions. Interestingly, the above research assumed that the adsorption effect occurs on the same side, and how about the opposite side? To this end, we explored the situation of 1O* and 1HO* when another side is also covered (Supplementary Fig. 1). We found that 2O* adsorption with each side adsorbing an O* can be thermodynamically favorable, which is somewhat similar to the observations of Svane et al. on SACs^27^. However, considering the bottom-side-O* adsorption does not change the general trends of surface Pourbaix diagrams because the EPW have little change compared to the 1O* adsorption at the front side. Given that adsorption at the bottom side could be kinetically less feasible under electrocatalytic conditions of some reactions and it also depends on the synthetic method of the catalyst (e.g., whether a large C-defect can form), we mainly analyzed the adsorption behavior at the front side in this paper.

### Adsorption of H*, O*, and HO* in a wide pH range

**Fig. 4:**
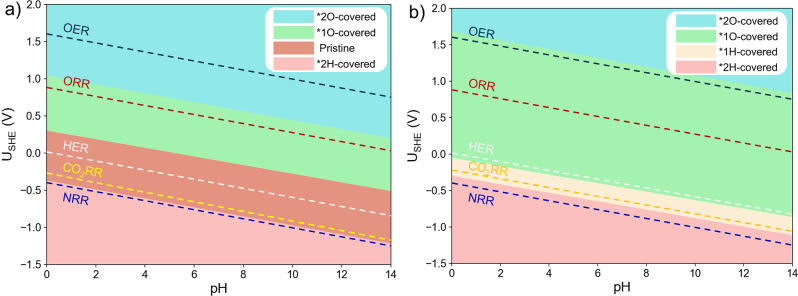
Examples of the surface Pourbaix diagrams as the function of pH and potential. **a** Surface Pourbaix diagrams in a wide pH range of Fe-Ni-C and **b** Fe-Ni-N_*1,2,4*_-C.

We further developed the surface Pourbaix diagrams of these models as the function of both potential and pH to fully explore the surface state evolution of DACs (Fig. 4 and Supplementary Table 3). The analysis of the representative Fe-Ni-C (Fig. 4a) and Fe-Ni-N_*1,2,4*_-C (Fig. 4b) models are elaborated below. Apparently, the diagrams are divided into several regions of different surface states. For Fe-Ni-C, we found that at *U*_SHE_ < −0.36 V, the active sites will be occupied by two 2H*. A pristine surface state can be retained within the EPW between −0.36 and 0.28 V. At higher potentials, the 1O* state becomes most favorable until *U*_SHE_ = 1.06 V, and further transits to the 2O* state when *U*_SHE_ is above 1.06 V. For the Fe-Ni-N_*1,2,4*_-C, we found that at U_SHE_ < −0.38 V, the active sites of this catalyst will be occupied by 2H*. At higher potentials, the active sites will be occupied by 1H*. When *U*_SHE_ > − 0.05 V, the 1O* state is thermodynamically most favorable. Due to these highly pH- and potential-dependent results, both operating potential and pH should be considered to determine the actual surface.

### Catalytic activity analysis using surface Pourbaix diagrams

The above analyses clearly show that the surface states of DACs are potential- and pH-dependent, suggesting that a DAC could exhibit a distinctive surface state at different electrocatalytic reaction conditions. We performed surface state analysis on the two-representative models discussed above at the characteristic potentials for various reactions. Considering the existence of overpotential in actual reaction, we employed experimental potentials which have been proven to be suitable as references, i.e., 1.60 V for oxygen evolution reaction (OER)^28^, 0.78 V for ORR^29^, 0 V for HER^30^, −0.35 V for CO_2_RR^31^, and −0.4 V for nitrogen reduction reaction (NRR)^32^. Because O* and HO* are also the key reaction intermediates of OER, we do not discuss the OER activities of DACs since whether the poisoning O* and HO* will participate in OER is still an open question. Likewise, how the adsorption of H* affects HER is not discussed here. Our calculations suggest that Fe-Ni-C (Fig. 4a) can offer a pristine surface at the CO_2_RR potential, making this reaction feasible. However, the occupation by 2H* at the NRR potential makes NRR less favorable. To sum up, Fe-Ni-C may be advantageous for CO_2_RR (at a moderate potential) but adverse for ORR and NRR. On the contrary, the EWP-unavailable Fe-Ni-N_*1,2,4*_-C model (Fig. 4b) will have the active sites covered by 1O* during ORR, 1H* during CO_2_RR, and fully occupied by 2H* at NRR potentials, hindering the reactions. Note that for CO_2_RR, the potential range is quite wide due to its numerous reaction types and products. Herein, only one typical reaction potential is discussed with surface Pourbaix diagram analyses. At more negative potentials, H will occupy the active sites for some models. These results are consistent with previous studies^26^.

**Fig. 5:**
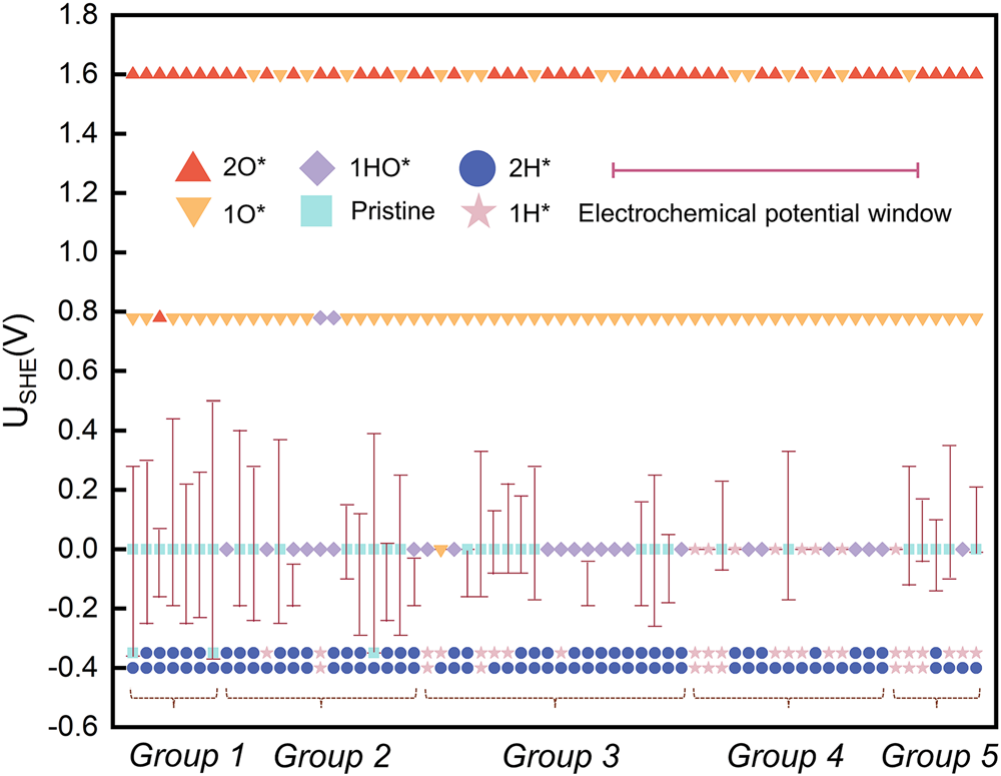
Surface states of Fe-Ni-N_*x*_-C at the common potentials of HER (0 V), OER (1.60 V), ORR (0.78 V), CO_2_RR (−0.35 V), and NRR (−0.4 V). Groups 1–5, respectively, indicate the number of coordinating N: 0–1, 2, 3, 4, and 5–6. The pink vertical lines represent the electrochemical potential window (EPW) of DACs and various symbols filled with different colors represent different occupying species. In detail, we employed the red upper triangle, orange lower triangle, purple diamond, cyan square, indigo circle, and pink star to describe DACs will be covered by 2O*, 1O*, 1OH*, pristine, 2H*, and 1H*, respectively, at the operating potentials.

We performed similar analyses on the other DACs (Fig. 5 and Supplementary Table 4). We found that almost all these catalysts will be covered by 1H* or 2H* at the NRR potential. Only a small number of DACs can retain the pristine state at the CO_2_RR potential, suggesting that a great majority of these 64 DACs may not be suitable for CO_2_RR. Fe-Ni-N_*1,2,3,4,5,6*_-C, a widely reported system in experimental synthesis^24,29^, would have its active sites occupied by 1H*. This phenomenon has been widely dismissed in previous studies (Fig. 1). Therefore, analyzing the surface state is particularly important before studying the electrochemical reaction on DACs. Note that in the present article, we do not analyze the electrocatalytic kinetics of these DACs but rather emphasize the availability of EPW as one of the important factors in DAC screening. For the DAC family, besides in-plane structures such as the systems analyzed in this paper, they can exit in a three-dimensional form (e.g., enzymes)^33^. These 3D DACs may exist in similar adsorption configurations compared with 2D DACs for some reactions^34^. We expect that our conclusions on DACs, as identified by the surface Pourbaix diagram, will also apply to other more complicated 3D DAC structures.

### A recommended DAC design framework for electrocatalysis

**Fig. 6:**
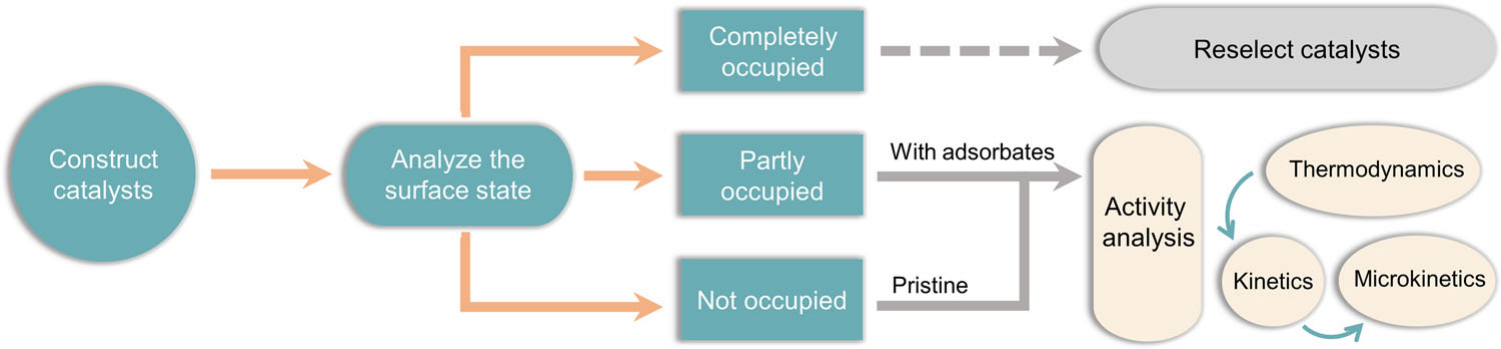
A recommended DAC design framework for electrocatalysis. A recommended framework for the design and analysis of DACs for electrocatalysis with external gas reactants (e.g., ORR, CO_2_RR, and NRR).

Based on the above discussions, a recommended theoretical analysis framework for designing DACs for electrocatalysis with external gas reactants (e.g., ORR, CO_2_RR, and NRR) is proposed in Fig. 6. The catalyst surface state and its equilibrium with the electrolyte should be analyzed before the study of electrochemical activity. The surface Pourbaix diagram analysis allows for identifying the thermodynamically favorable surface states at the potentials of interest. Subsequently, more accurate activity analysis (e.g., reaction kinetic and thermodynamic calculations, and microkinetic modeling) should be performed based upon the most favorable surface state at the operating potentials. Besides, in-situ surface state analysis methods, such as infrared, Raman, X-ray photoelectron, and X-ray absorption spectroscopy, should be applied to provide experimentally-determined surface information for accurate surface probing.

## Conclusion

In summary, using spin-polarized DFT-D3 analysis, we show that the surface states of a series of DAC (M-M’-N_*x*_-C) models generally differ from a pristine state at the operating conditions for several important electrocatalytic reactions. The strong adsorption capability of the metal-metal bridge site in DACs may attract atoms or molecules from the liquid dissolvent to adsorb. This finding suggests that electrochemical-driven pre-adsorption of molecules may block the active site of DACs. Therefore, the surface state of a DAC should be analyzed before the detailed catalytic activity analysis, where exploration of the surface Pourbaix diagram is essential for the subsequent analysis of the electrocatalytic mechanism.

## Methods

### Surface Pourbaix diagrams

Surface Pourbaix diagrams reveal the surface states as the function of pH and potential, providing a thermodynamic indication of whether the catalyst will be poisoned. Considering DACs pristine surface (P) with the adsorption site (*) and the pre-adsorbed molecule (O_*m*_H_n_*), the adsorption equation can be written as:

3 $${{{{{\rm{P}}}}}}-{O}_{m}{H}_{n}^{\ast }+(2m-n)({{{{{\rm{e}}}}}}^{-}+{{{{{{\rm{H}}}}}}}^{+})\iff {{{{{{\rm{P}}}}}}}^{\ast }+m{{{{{{\rm{H}}}}}}}_{2}{{{{{\rm{O}}}}}}$$

where *m* and *n* are, respectively, the number of oxygen and hydrogen atoms of the adsorbate. The free energy changes were calculated using:

4 $$\Delta G={G}_{{{{{{{\rm{P}}}}}}}^{\ast }}+m{G}_{{{{{{{\rm{H}}}}}}}_{2}{{{{{\rm{O}}}}}}}-{G}_{{{{{{\rm{P}}}}}}-{{{{{{\rm{O}}}}}}}_{{m}}{{{{{{\rm{H}}}}}}}_{{n}}^{\ast }}-(2m-n)(0.5{G}_{{{{{{{\rm{H}}}}}}}_{2}}-{U}_{{{{{{\rm{SHE}}}}}}}-2.303\,{{k}}_{{{{{{{\rm{B}}}}}}}}T{{{{{\rm{pH}}}}}})$$

where *U*_SHE_ is the potential relative to the standard hydrogen electrode (SHE), and *k*_B_ is the Boltzmann constant (8.617343 × 10^–5^ eV K^–1^).

### Computational details

In this work, all DFT calculations were performed using the Vienna ab initio simulation package (VASP 5.4.4) with the Perdew–Burke–Ernzerhof (PBE) functional and projector augmented wave (PAW) potentials^35,36^, which have been proven suitable for graphene-based materials^37^. The impact of spin-polarization and van der Waals dispersion were included in all calculations^38,39^. For the dual-atom electrocatalyst model, a 5 × √3 graphene with a vacuum layer of 20 Å was used to simulate the catalyst surface. In structural optimization, a 2 × 2 × 1 Γ-centered *k*-point mesh grid and 450 eV cutoff energy were adopted. The convergence standards of energy and force were set to 10^−5^ eV and 0.02 eV/Å, respectively. To obtain more accurate results, a 4 × 4 × 1 Γ-centered *k*-point mesh grid was adopted for the subsequent self-consistent field calculations. The Gibbs free energies (Δ*G*) were calculated using the following equation^40^:

5 $$\Delta G=\Delta E+\Delta {{{{{\rm{ZPE}}}}}}-T\Delta S$$

where Δ*E* is the difference of electronic energy in the ground state obtained from self-consistent calculations, ΔZPE is the difference of zero-point energies, *T* is the temperature (298.15 K), and Δ*S* is the difference in entropy. The entropies of molecules in the gas phase were taken from the NIST database.

## Supplementary information

Supplementary Information

## Data Availability

All related data and optimized geometries are stored in https://github.com/dualatoms/surface_pourbaix. All data presented in this study are available from the corresponding authors upon request.
