# Supplementary material for: Surface states of dual-atom catalysts should be considered for analysis of electrocatalytic activity
Source: Commun Chem. 2023 Jan 6;6:6. doi: 10.1038/s42004-022-00810-4 (PMC9822963; doi:10.1038/s42004-022-00810-4)
Supplement: Supplementary file 1 — Supplementary Information [file 42004_2022_810_MOESM1_ESM.pdf]

Supplementary Information

Surface states of dual-atom catalysts should be considered for analysis of electrocatalytic activity

Weijie Yang[1], Zhenhe Jia[1], Binghui Zhou[1], Li Wei[2], Zhengyang Gao[1 *], Hao Li[3 *]

[1] Department of Power Engineering, School of Energy, Power and Mechanical Engineering, North China Electric Power University, Baoding 071003, China

[2] School of Chemical and Biomolecule Engineering, The University of Sydney, Darlington 2006, NSW, Australia

[3] Advanced Institute for Materials Research (WPI-AIMR), Tohoku University, Sendai 980-8577, Japan

*** e-mail:** gaozhyan@163.com; li.hao.b8@tohoku.ac.jp

Supplementary Table 1. Surface Pourbaix diagrams of 3*d* homonuclear DACs at pH = 0.

Ti_2-$N_{1,2,3,4,5,6}$-C

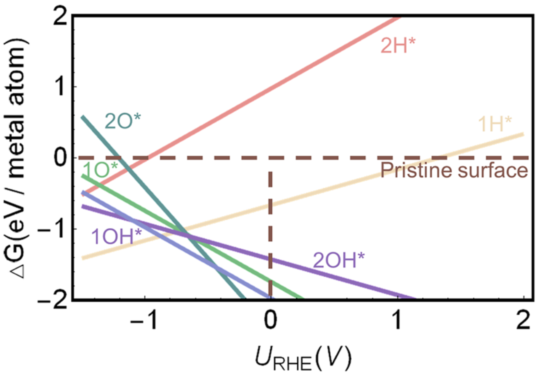

V_2-$N_{1,2,3,4,5,6}$-C

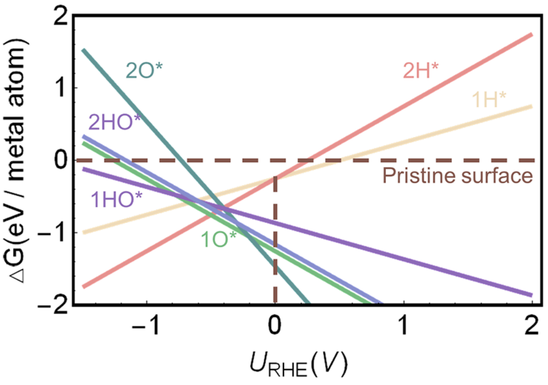

Cr_2-$N_{1,2,3,4,5,6}$-C

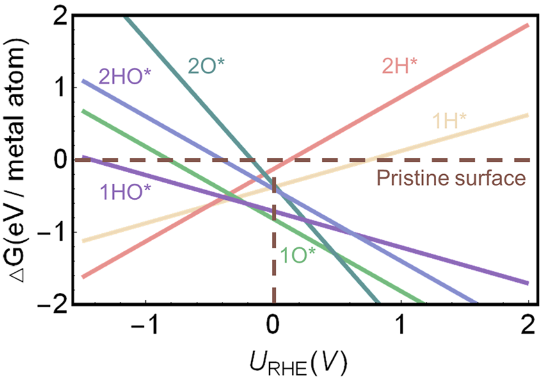

Mn_2-$N_{1,2,3,4,5,6}$-C

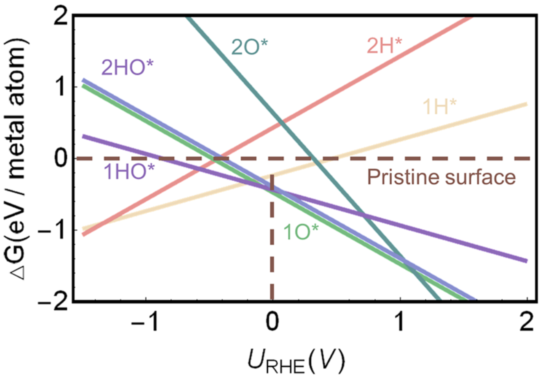

Fe_2-$N_{1,2,3,4,5,6}$-C

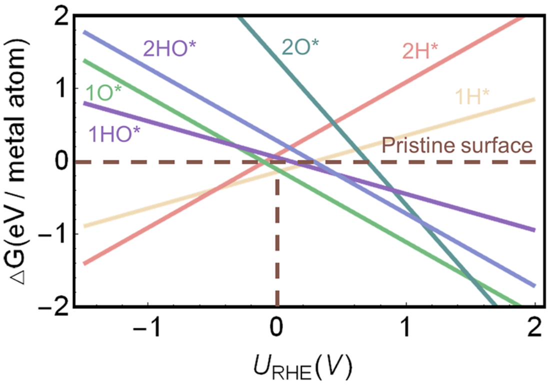

Co_2-$N_{1,2,3,4,5,6}$-C

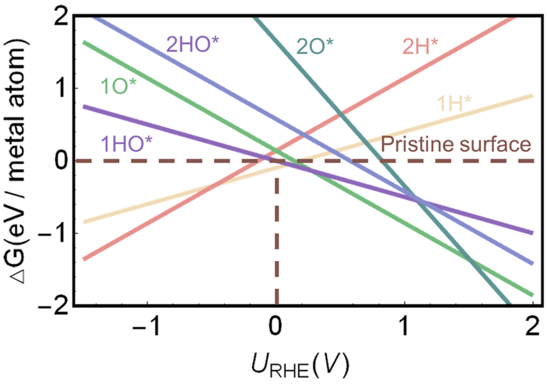

Ni_2-$N_{1,2,3,4,5,6}$-C

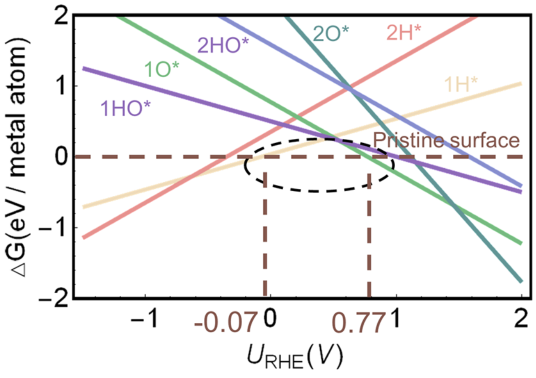

Cu_2-$N_{1,2,3,4,5,6}$-C

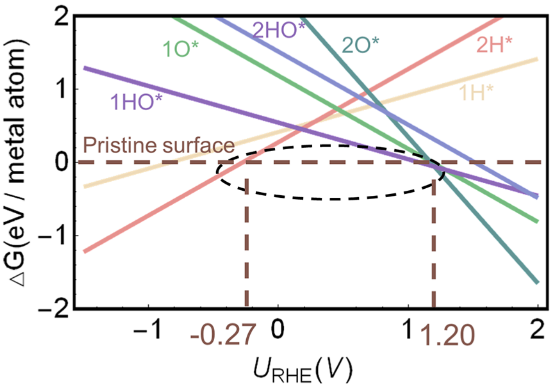

Supplementary Table 2. Surface Pourbaix diagrams of Fe-Ni-N_x-C at pH = 0

Fe-Ni-C

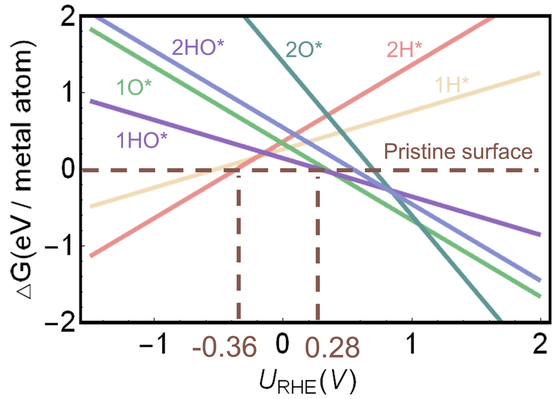

Fe-Ni-N_1-C

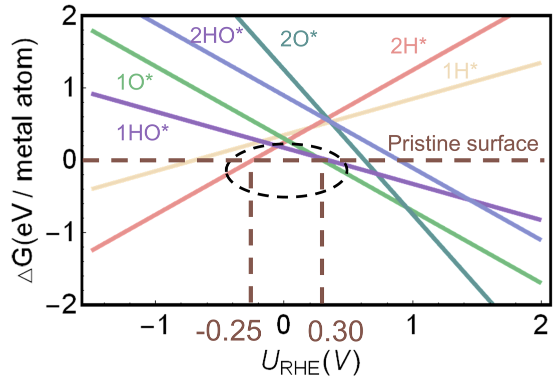

Fe-Ni-N_2-C

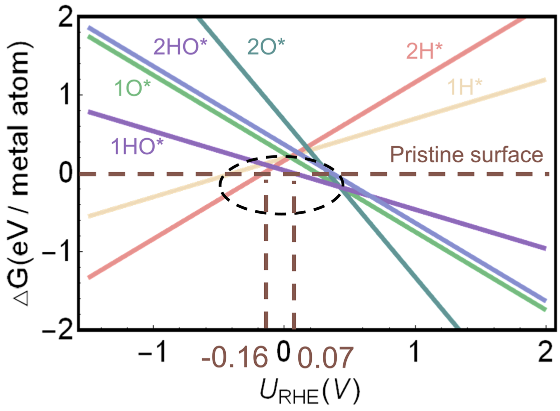

Fe-Ni-N_3-C

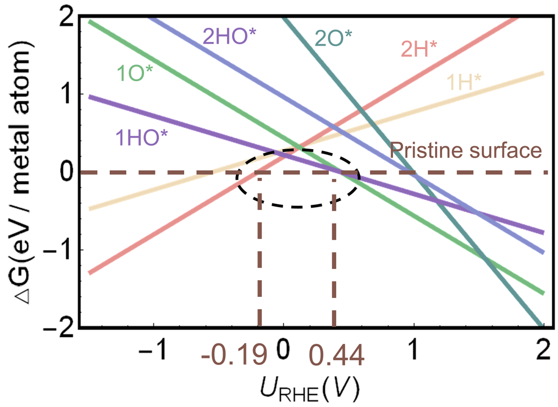

Fe-Ni-N_4-C

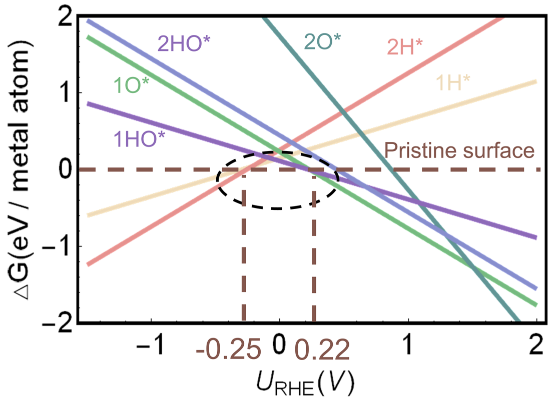

Fe-Ni-N_5-C

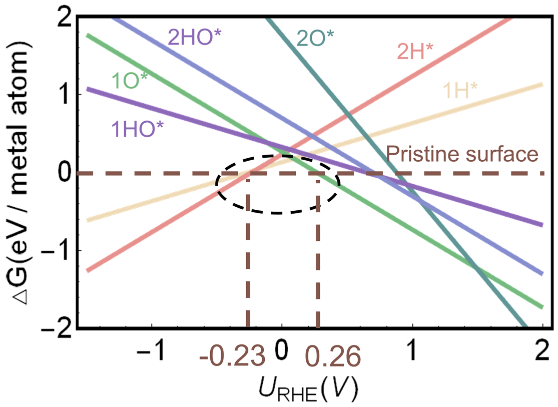

Fe-Ni-N_6-C

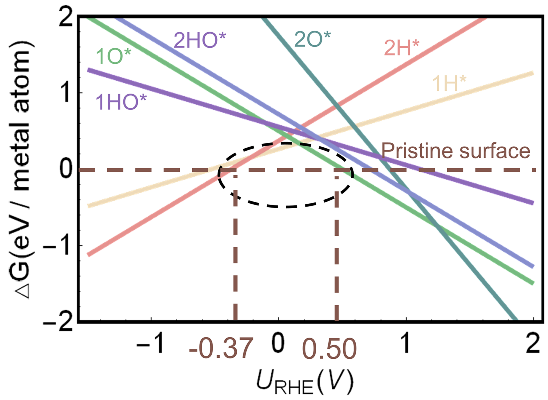

Fe-Ni-$N_{1,2}$-C

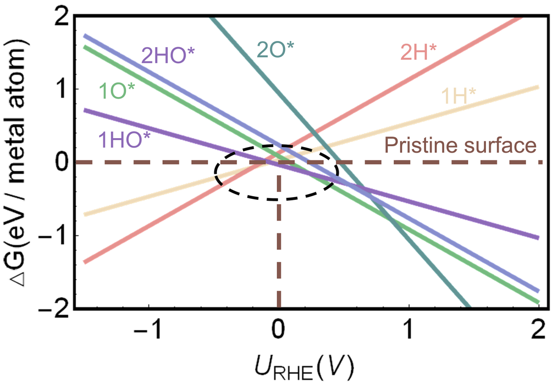

Fe-Ni-$N_{1,3}$-C	Fe-Ni-$N_{1,4}$-C
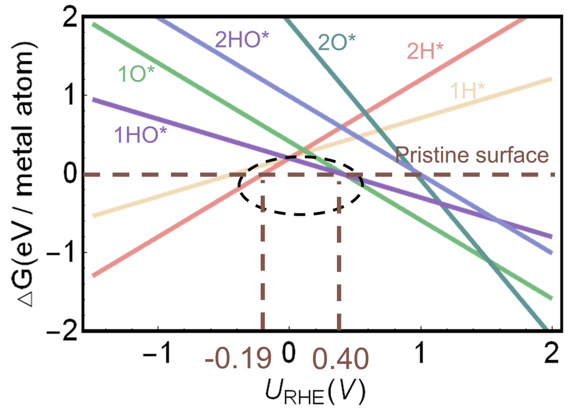	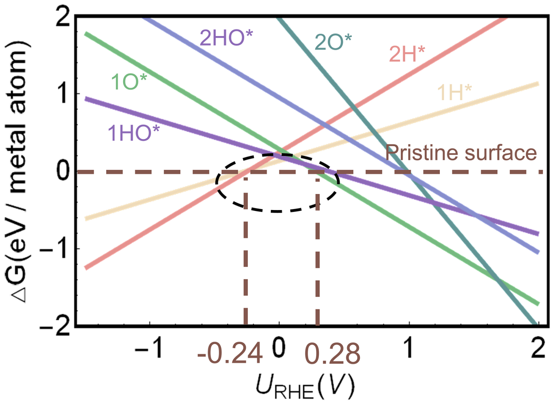

Fe-Ni-$N_{1,5}$-C	Fe-Ni-$N_{1,6}$-C
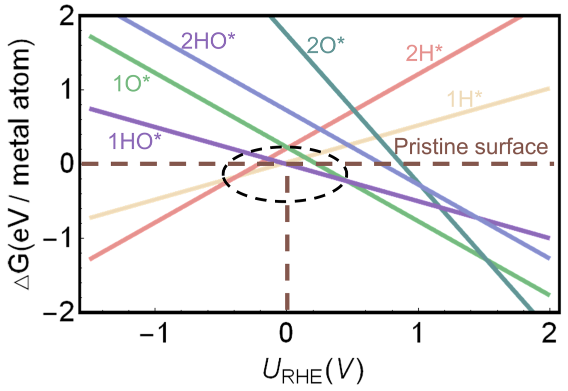	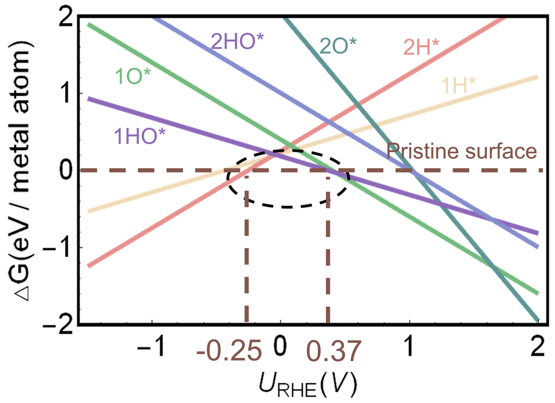

Fe-Ni-$N_{2,3}$-C	Fe-Ni-$N_{2,4}$-C
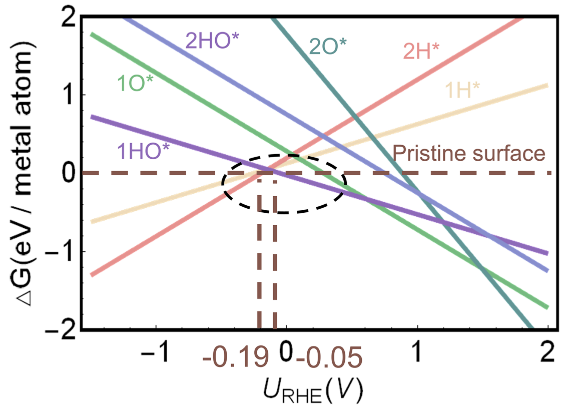	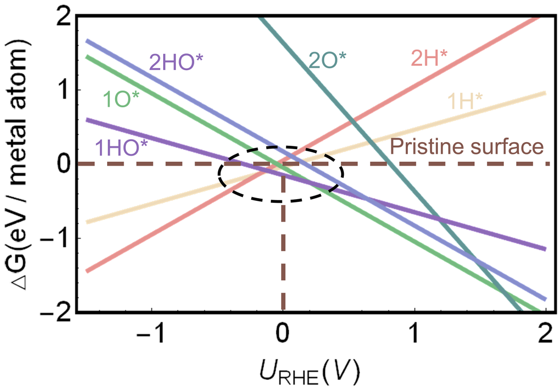

Fe-Ni-$N_{2,5}$-C	Fe-Ni-$N_{2,6}$-C
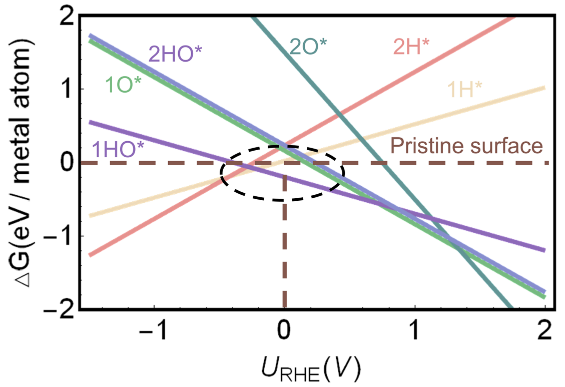	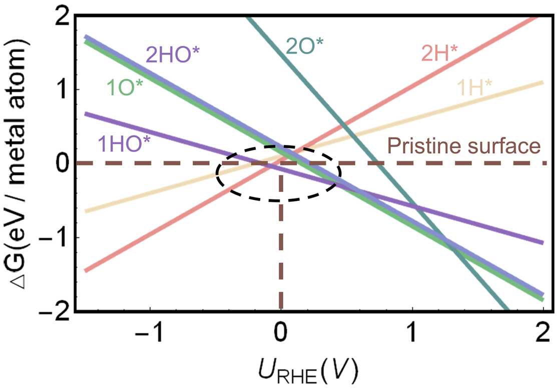

Fe-Ni-$N_{3,4}$-C

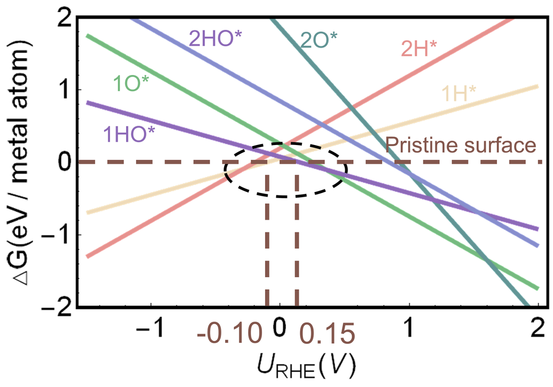

Fe-Ni-$N_{3,5}$-C

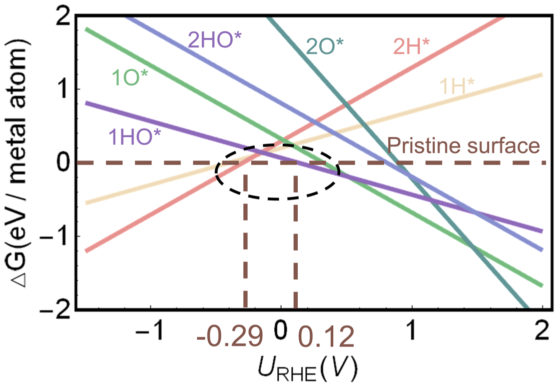

Fe-Ni-$N_{3,6}$-C

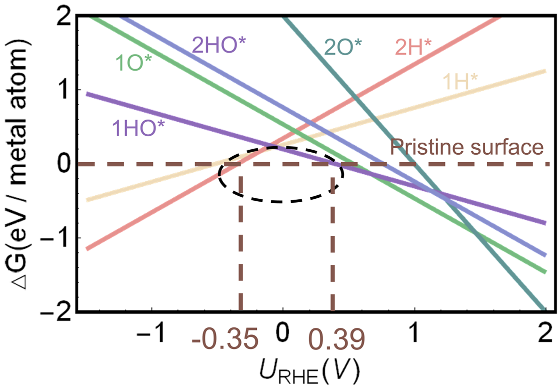

Fe-Ni-$N_{4,5}$-C

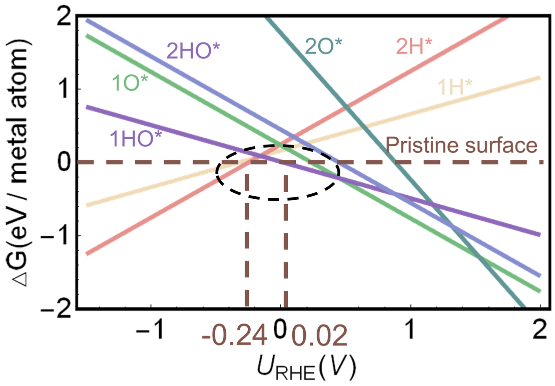

Fe-Ni-$N_{4,6}$-C

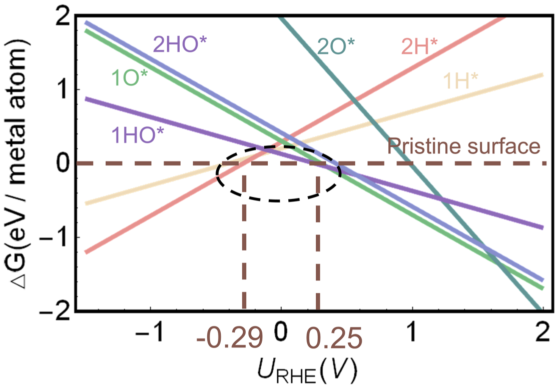

Fe-Ni-$N_{5,6}$-C

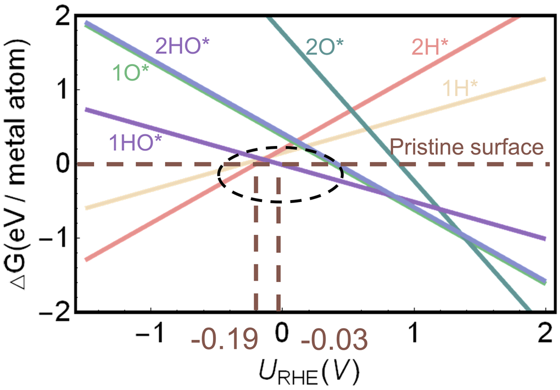

Fe-Ni-$N_{1,2,3}$-C

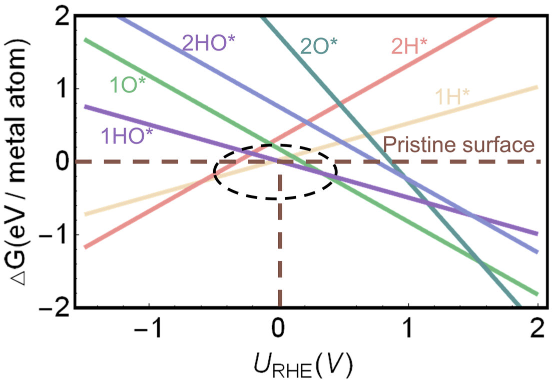

Fe-Ni-$N_{1,2,4}$-C

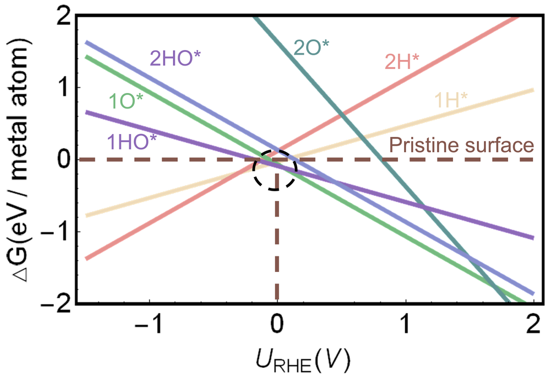

Fe-Ni-N$_{1,2,5}$-C	Fe-Ni-N$_{1,2,6}$-C
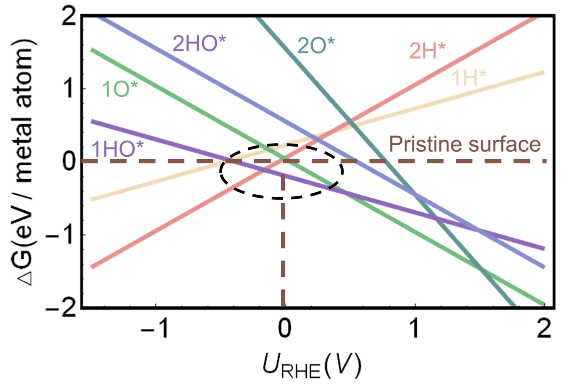	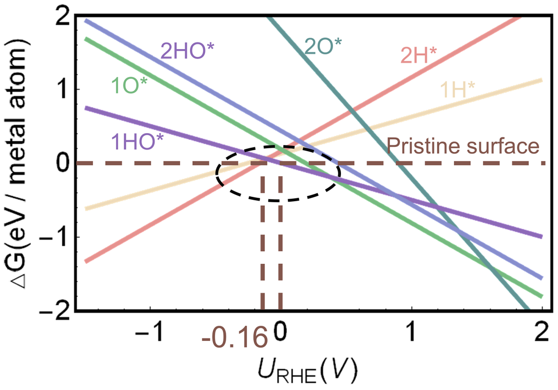

Fe-Ni-N$_{1,3,4}$-C	Fe-Ni-N$_{1,3,5}$-C
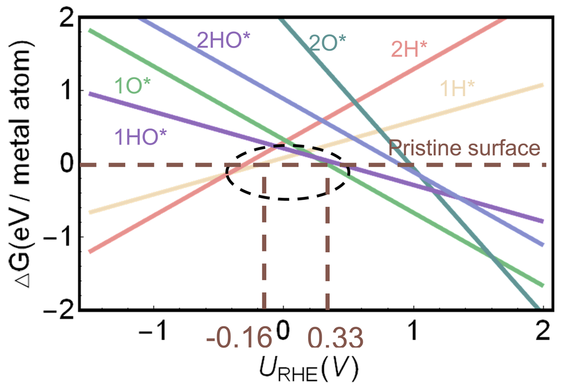	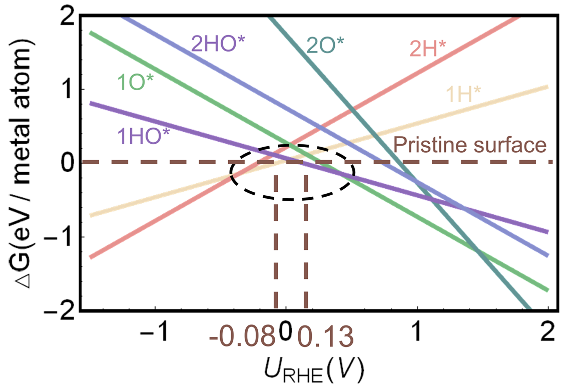

Fe-Ni-N$_{1,3,6}$-C	Fe-Ni-N$_{1,4,5}$-C
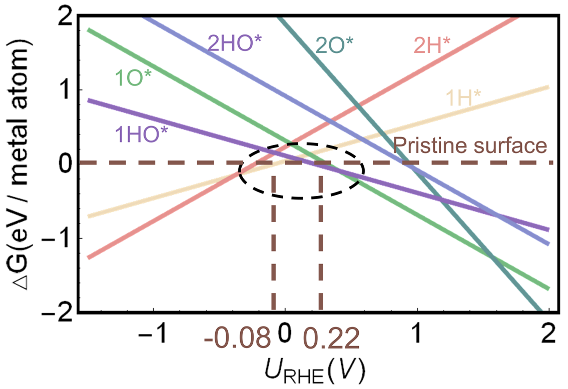	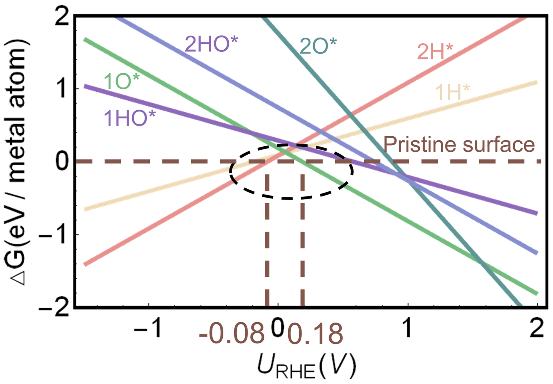

Fe-Ni-N$_{1,4,6}$-C	Fe-Ni-N$_{1,5,6}$-C
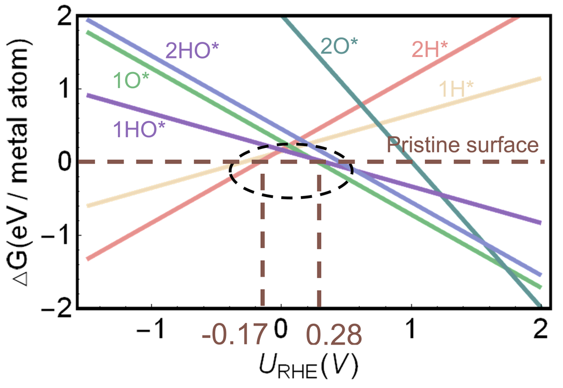	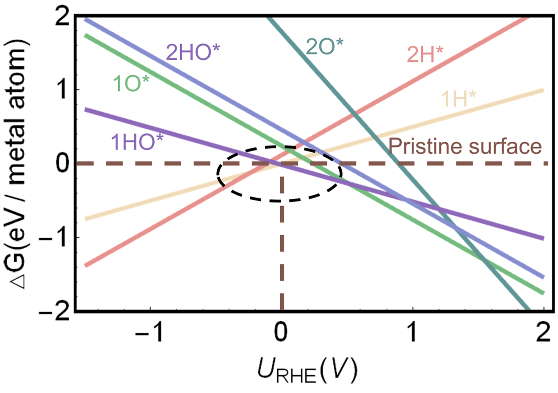

Fe-Ni-$N_{2,3,4}$-C

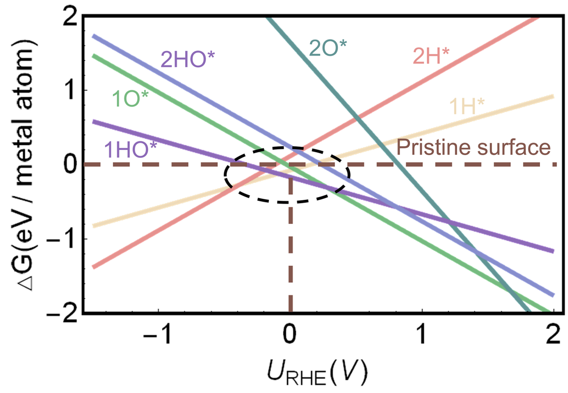

Fe-Ni-$N_{2,3,5}$-C

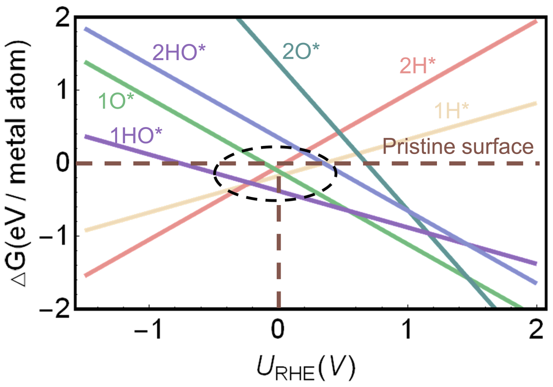

Fe-Ni-$N_{2,3,6}$-C

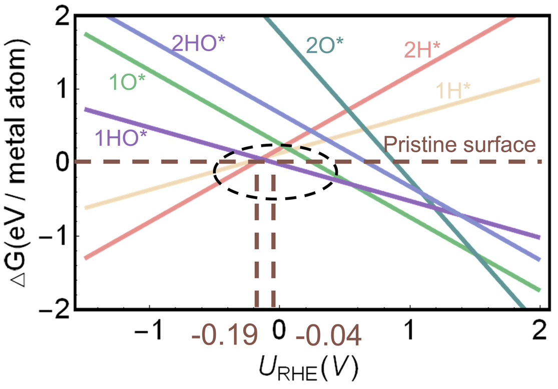

Fe-Ni-$N_{2,4,5}$-C

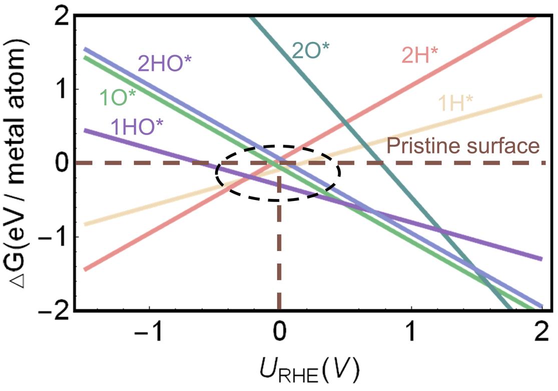

Fe-Ni-$N_{2,4,6}$-C

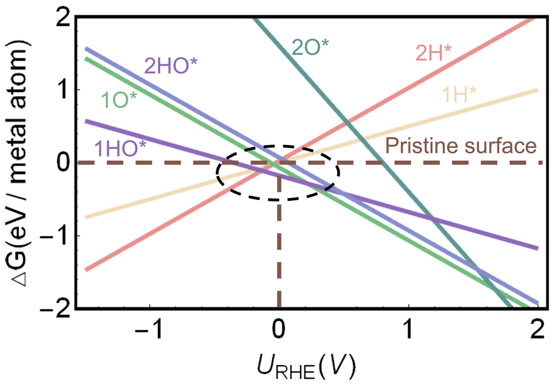

Fe-Ni-$N_{2,5,6}$-C

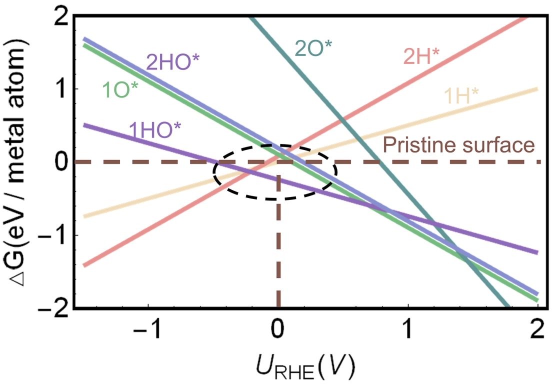

Fe-Ni-$N_{3,4,5}$-C

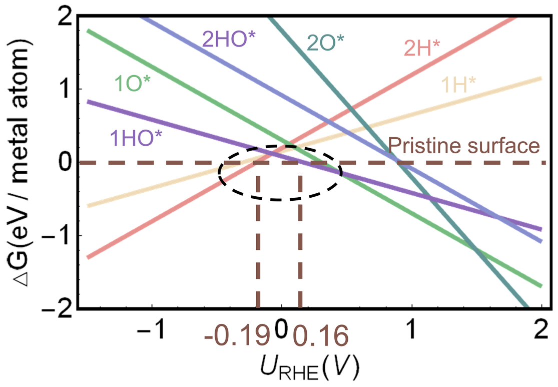

Fe-Ni-$N_{3,4,6}$-C

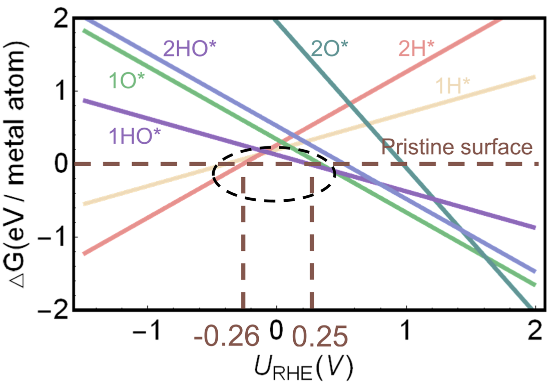

Fe-Ni-$N_{3,5,6}$-C	Fe-Ni-$N_{4,5,6}$-C
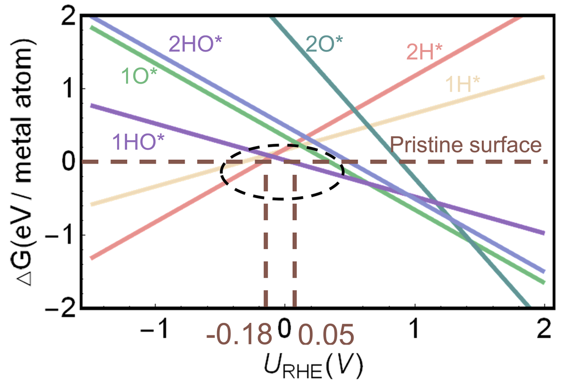	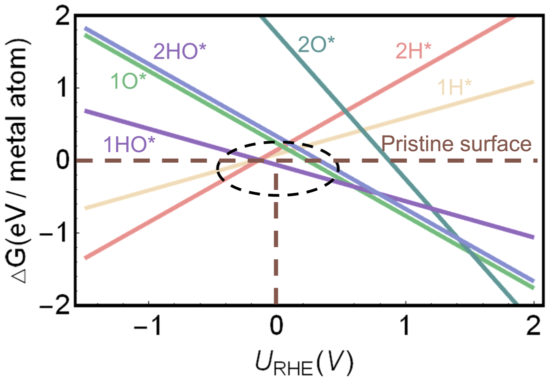

Fe-Ni-$N_{1,2,3,4}$-C	Fe-Ni-$N_{1,2,3,5}$-C
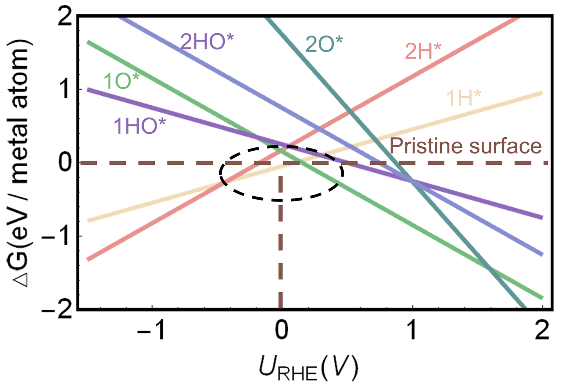	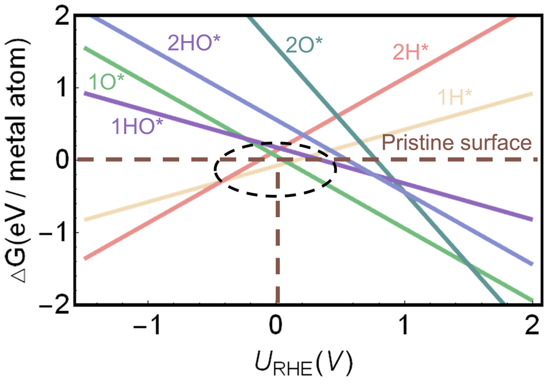

Fe-Ni-$N_{1,2,3,6}$-C	Fe-Ni-$N_{1,2,4,5}$-C
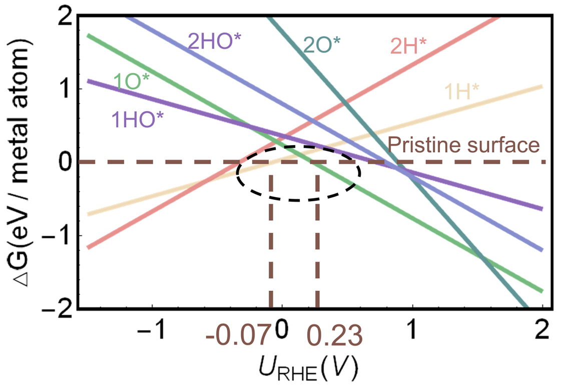	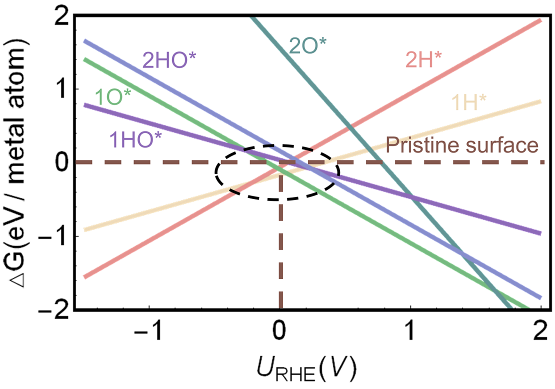

Fe-Ni-$N_{1,2,4,6}$-C	Fe-Ni-$N_{1,2,5,6}$-C
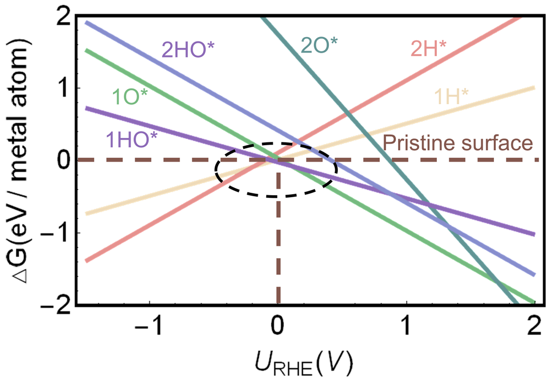	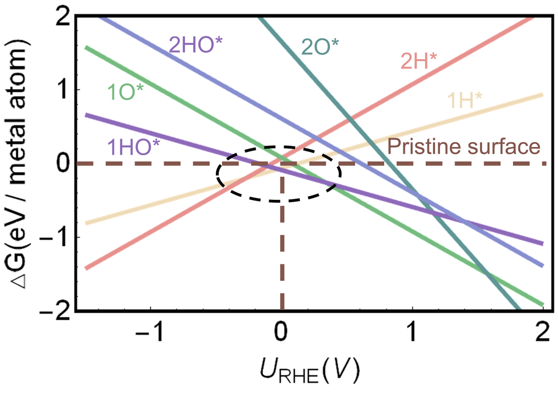

Fe-Ni-N$_{1,3,4,5}$-C	Fe-Ni-N$_{1,3,4,6}$-C

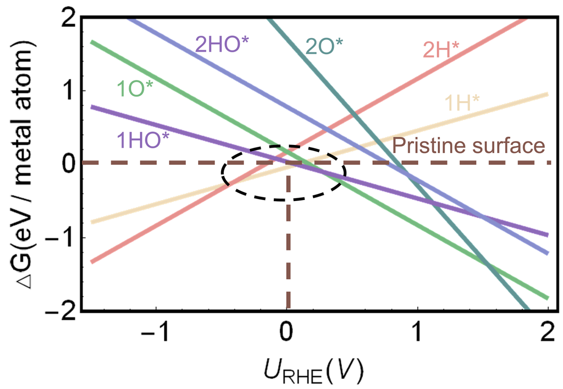

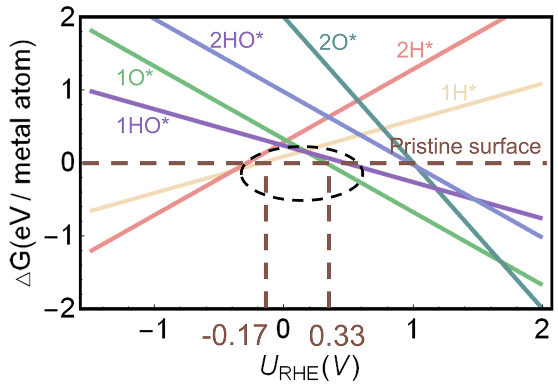

Fe-Ni-N$_{1,3,5,6}$-C	Fe-Ni-N$_{1,4,5,6}$-C

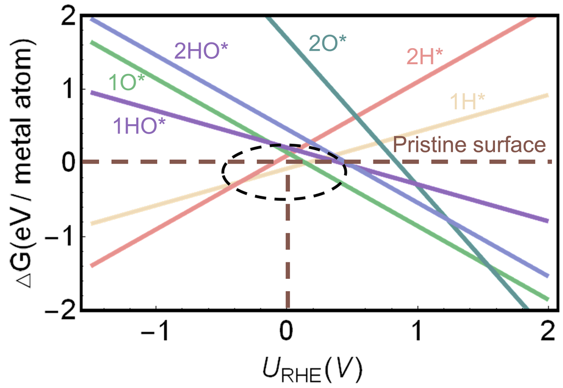

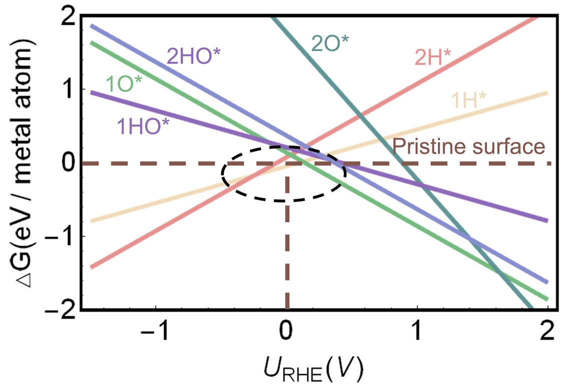

Fe-Ni-N$_{2,3,4,5}$-C	Fe-Ni-N$_{2,3,4,6}$-C

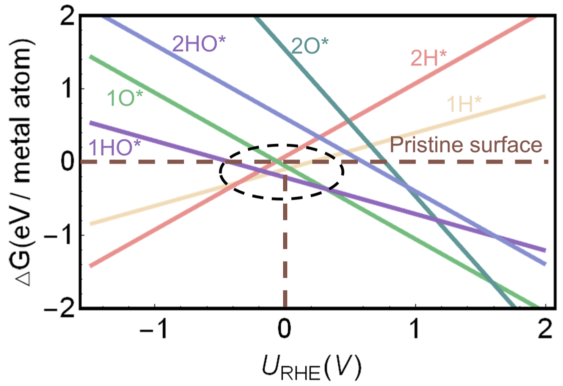

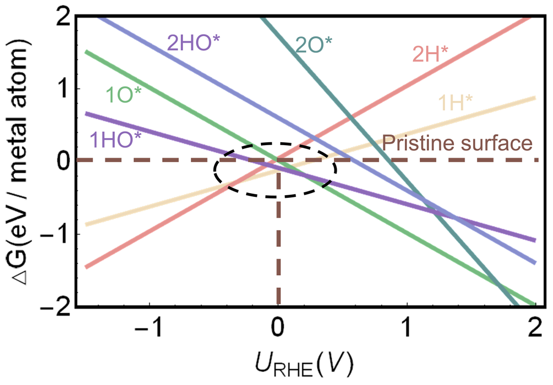

Fe-Ni-N$_{2,3,5,6}$-C	Fe-Ni-N$_{2,4,5,6}$-C

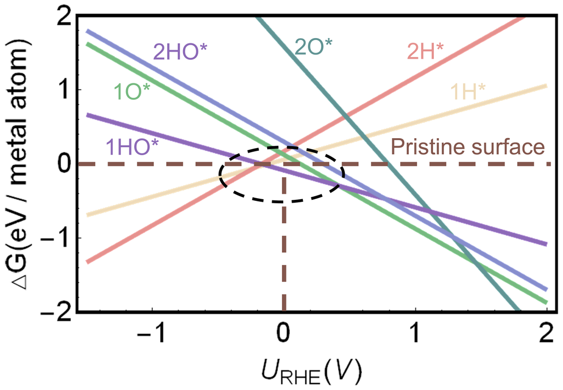

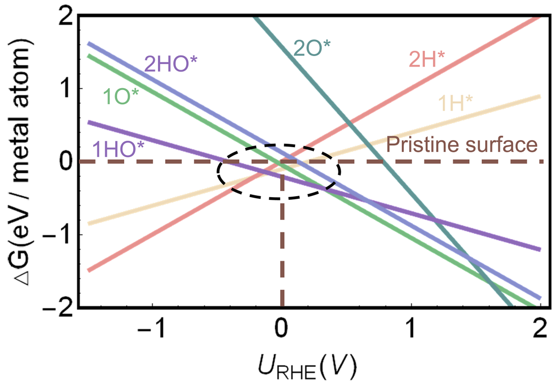

Fe-Ni-N$_{3,4,5,6}$-C

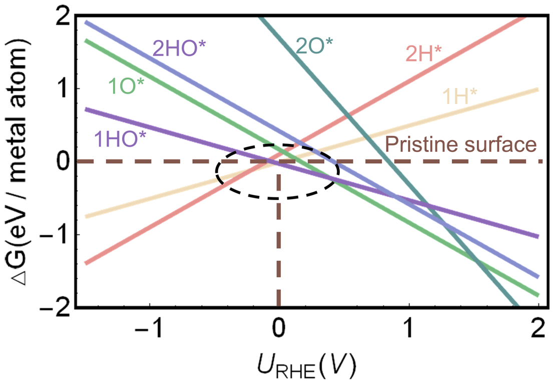

Fe-Ni-N$_{1,2,3,4,5}$-C

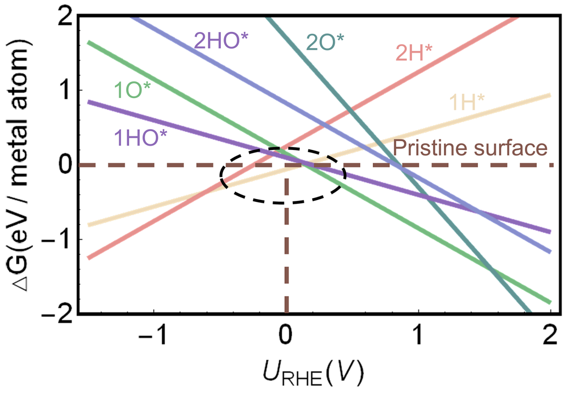

Fe-Ni-N$_{1,2,3,4,6}$-C

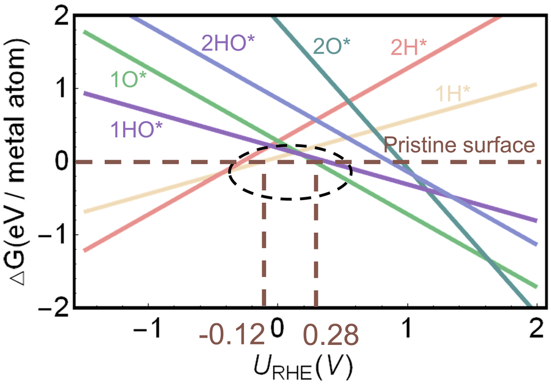

Fe-Ni-N$_{1,2,3,5,6}$-C

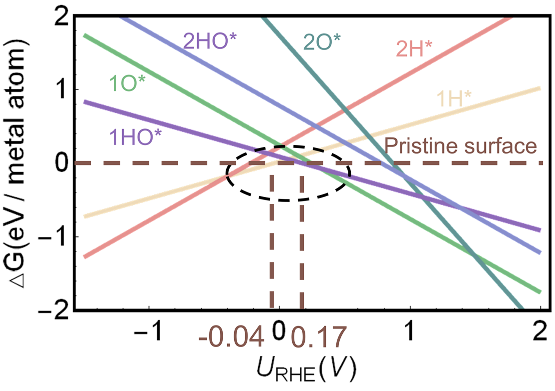

Fe-Ni-N$_{1,2,4,5,6}$-C

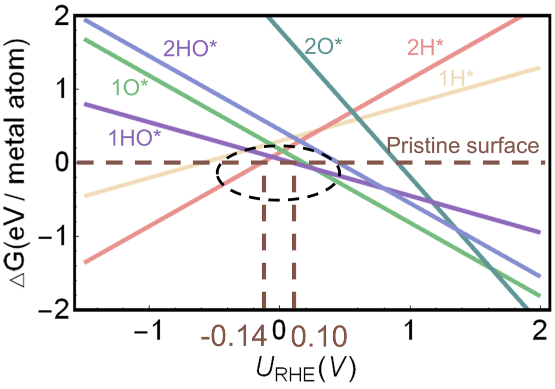

Fe-Ni-N$_{1,3,4,5,6}$-C

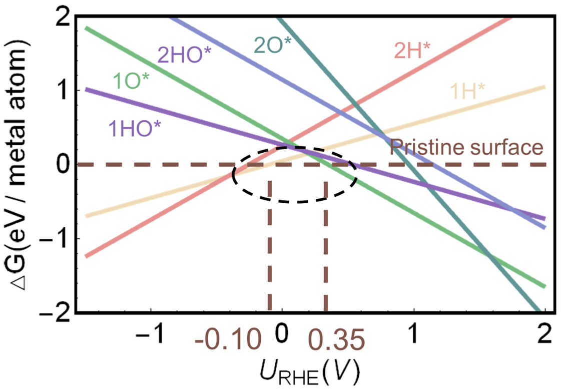

Fe-Ni-N$_{2,3,4,5,6}$-C

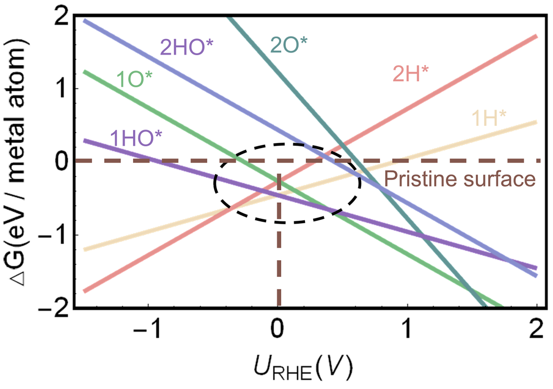

Fe-Ni-N$_{1,2,3,4,5,6}$-C

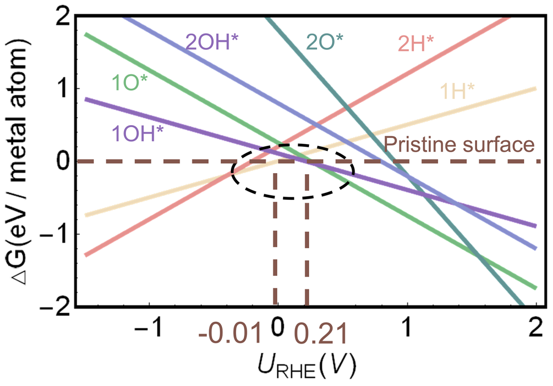

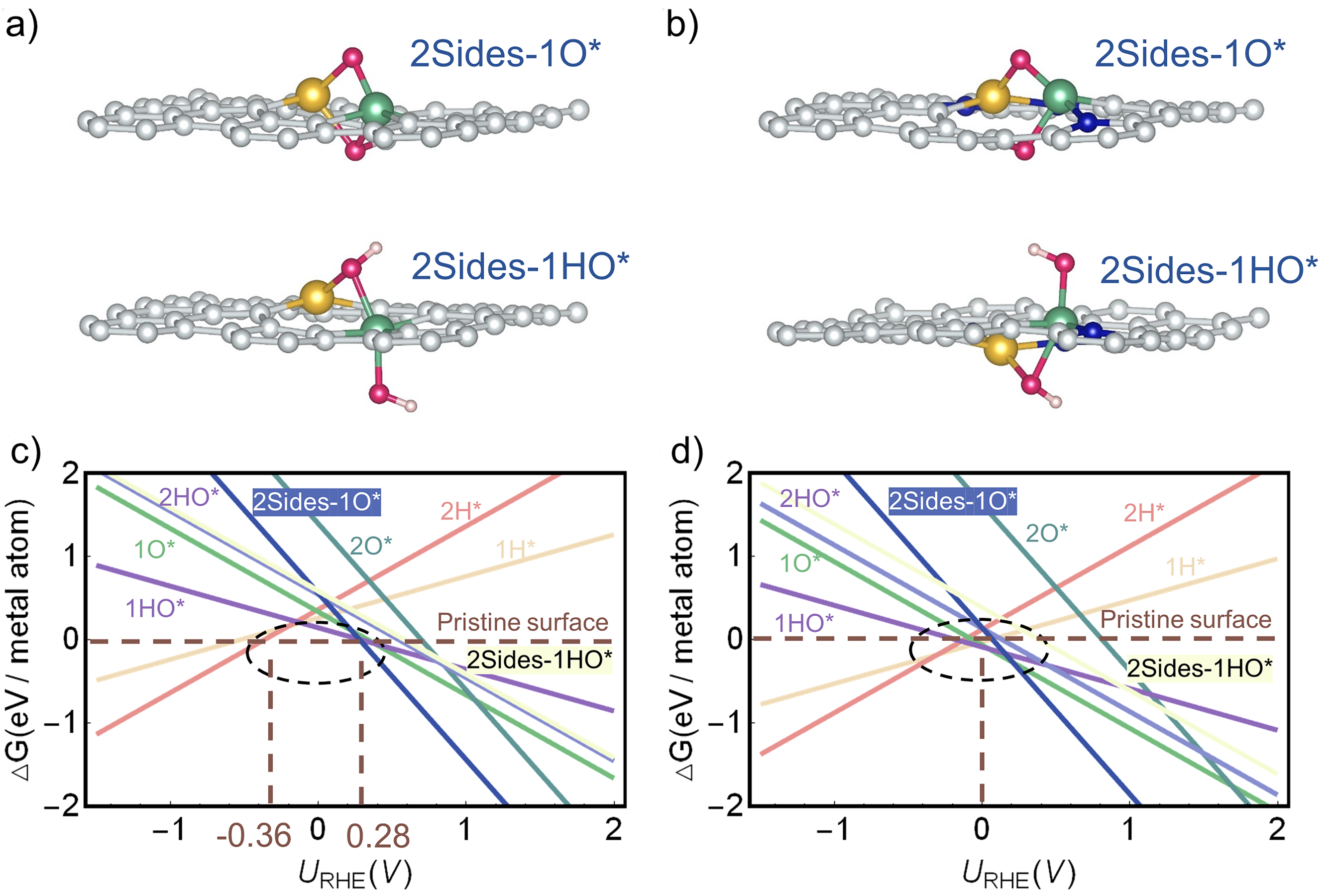

Supplementary Figure 1. Examples of the surface Pourbaix diagrams of DACs at pH=0. a) Two-side adsorption configurations of Fe-Ni-C, **b)** and Fe-Ni-$N_{1,2,4}$-C, where yellow, green, blue, silvery, red, and light pink spheres represent Ni, Fe, N, C, O, and H respectively. **c)** The surface Pourbaix diagrams of Fe-Ni-C, **d)** and Fe-Ni-$N_{1,2,4}$-C.

Supplementary Table 3. Surface Pourbaix diagrams of Fe-Ni-N$_x$-C in both acid and base.

Fe-Ni-C	Fe-Ni-N$_{1}$-C
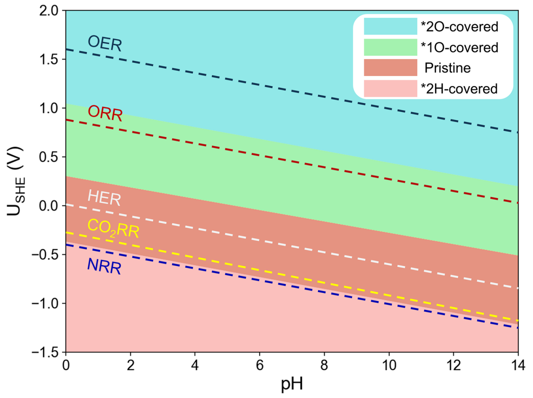	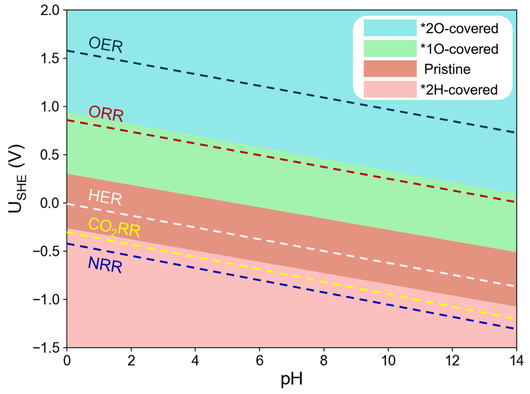
**Fe-Ni-N$_{2}$-C**	**Fe-Ni-N$_{3}$-C**
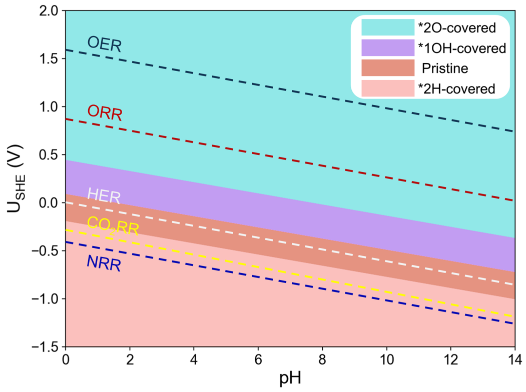	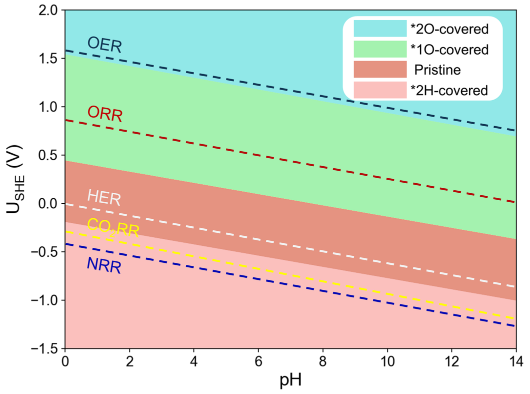
**Fe-Ni-N$_{4}$-C**	**Fe-Ni-N$_{5}$-C**
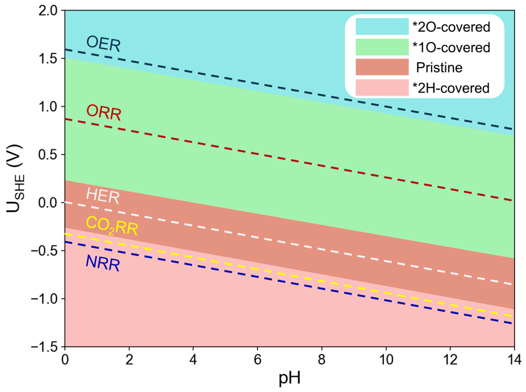	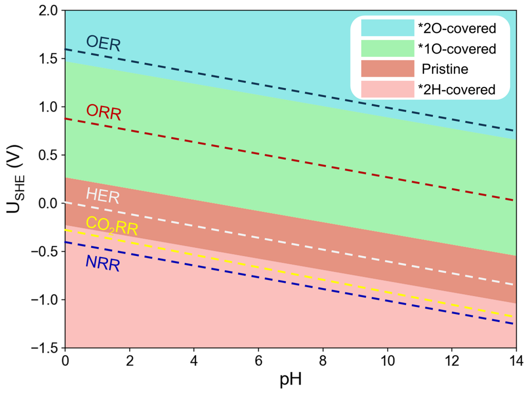
**Fe-Ni-N$_{6}$-C**	**Fe-Ni-N$_{1,2}$-C**
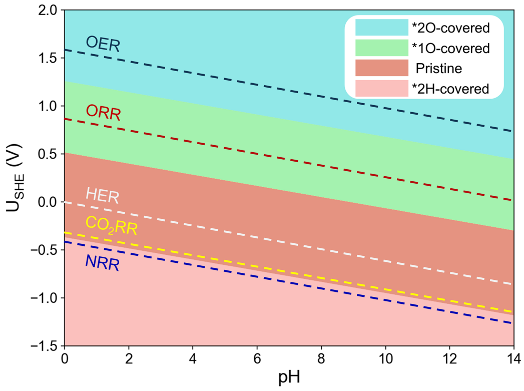	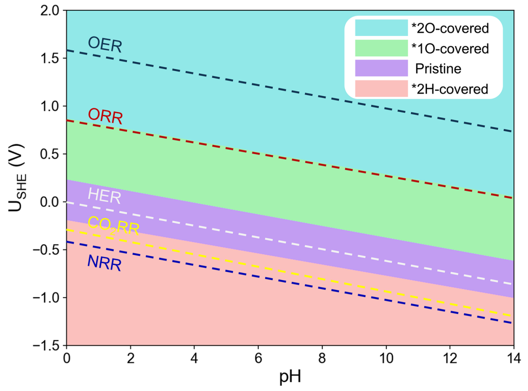

Fe-Ni-$N_{1,3}$-C

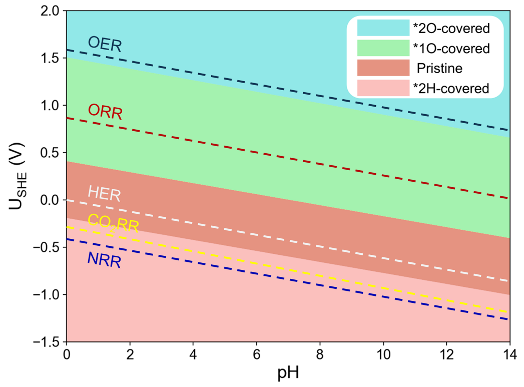

Fe-Ni-$N_{1,4}$-C

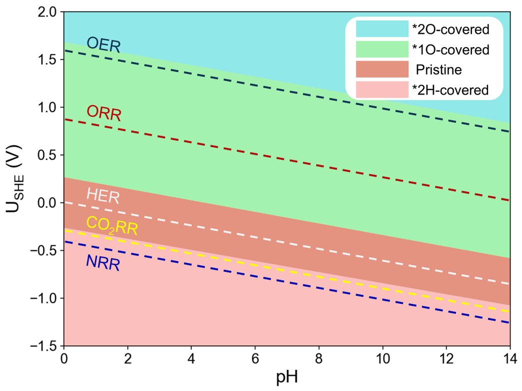

Fe-Ni-$N_{1,5}$-C

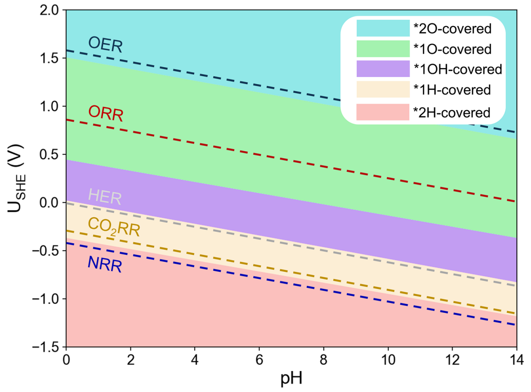

Fe-Ni-$N_{1,6}$-C

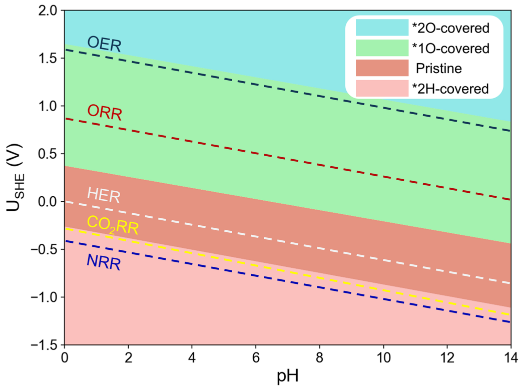

Fe-Ni-$N_{2,3}$-C

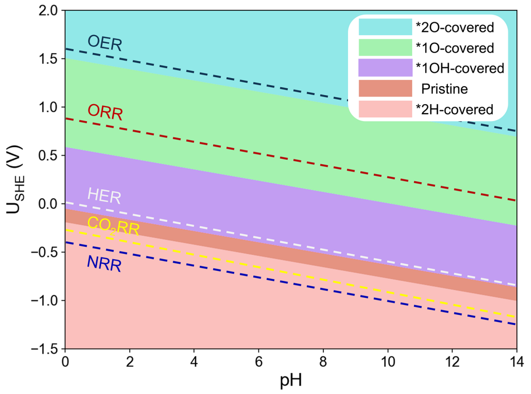

Fe-Ni-$N_{2,4}$-C

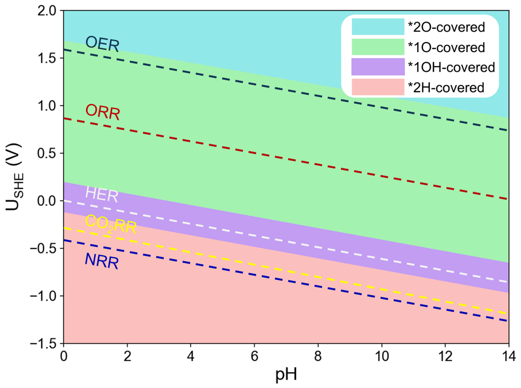

Fe-Ni-$N_{2,5}$-C

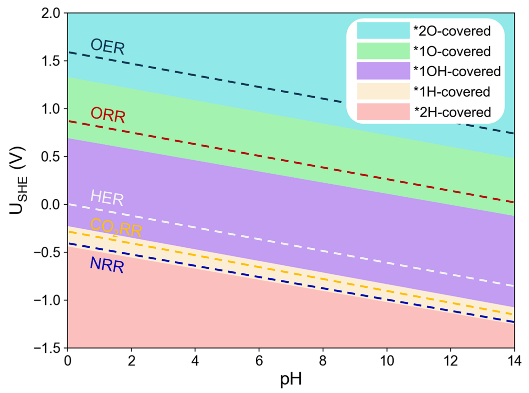

Fe-Ni-$N_{2,6}$-C

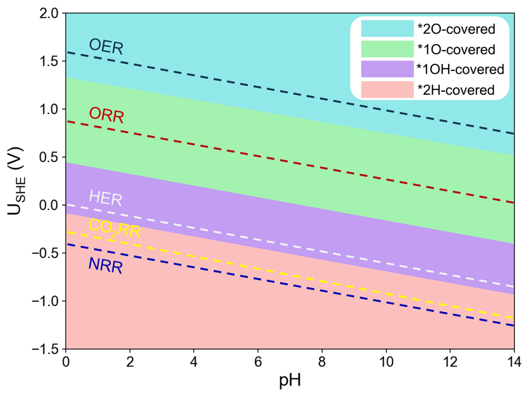

Fe-Ni-N$_{3,4}$-C	Fe-Ni-N$_{3,5}$-C

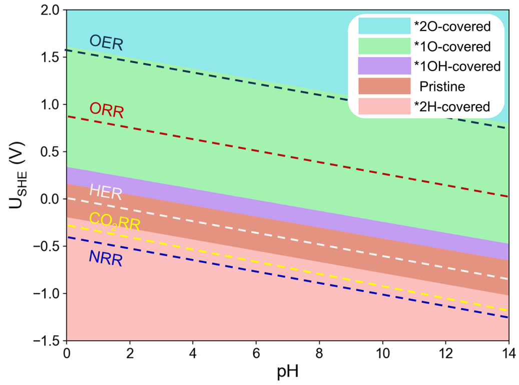

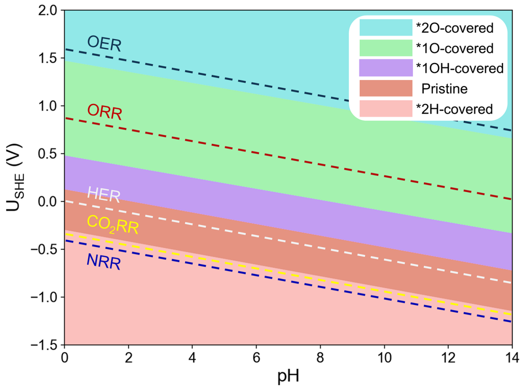

Fe-Ni-N$_{3,6}$-C	Fe-Ni-N$_{4,5}$-C

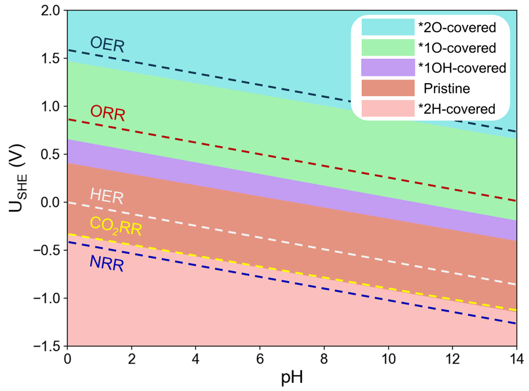

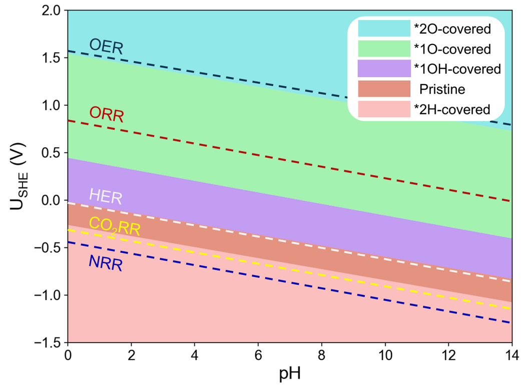

Fe-Ni-N$_{4,6}$-C	Fe-Ni-N$_{5,6}$-C

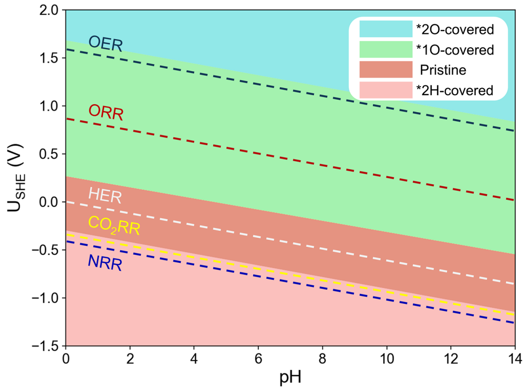

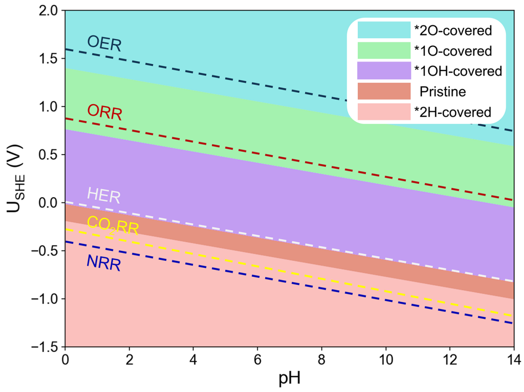

Fe-Ni-N$_{1,2,3}$-C	Fe-Ni-N$_{1,2,4}$-C

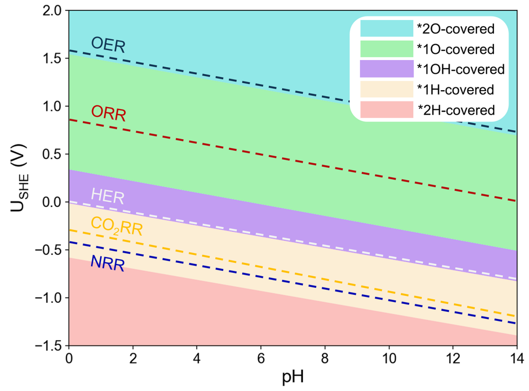

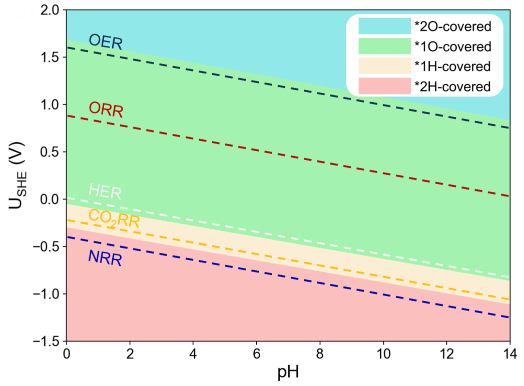

Fe-Ni-N$_{1,2,5}$-C

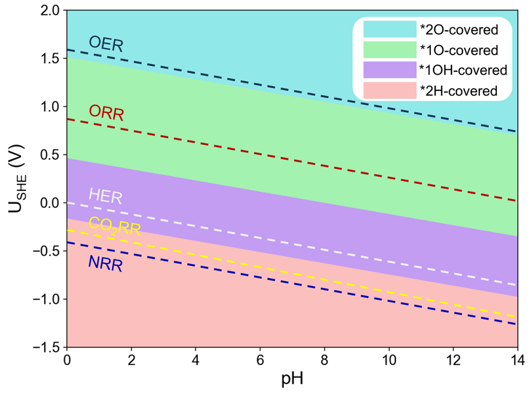

Fe-Ni-N$_{1,2,6}$-C

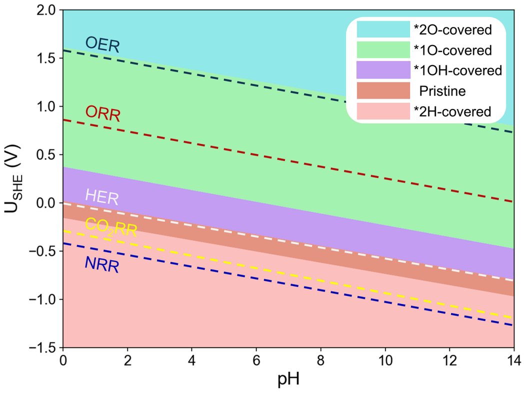

Fe-Ni-N$_{1,3,4}$-C

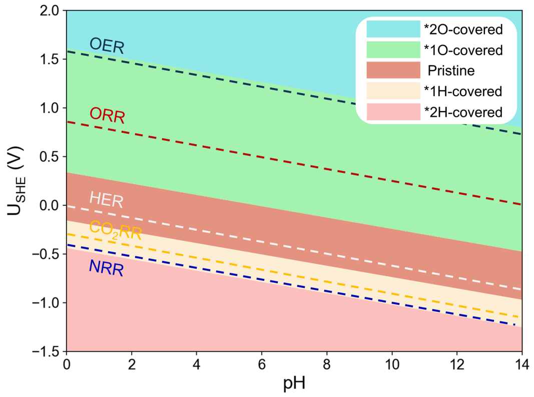

Fe-Ni-N$_{1,3,5}$-C

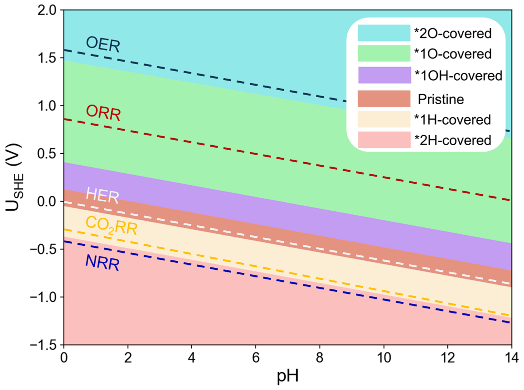

Fe-Ni-N$_{1,3,6}$-C

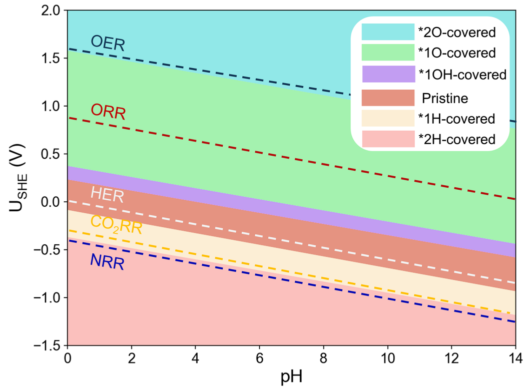

Fe-Ni-N$_{1,4,5}$-C

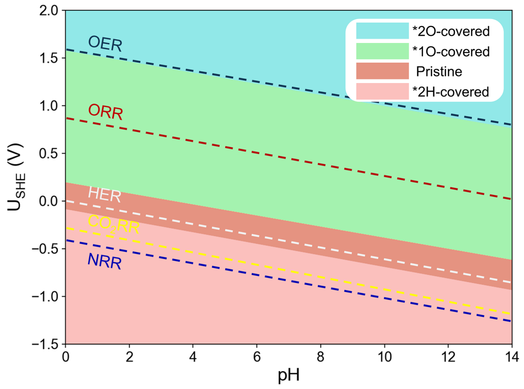

Fe-Ni-N$_{1,4,6}$-C

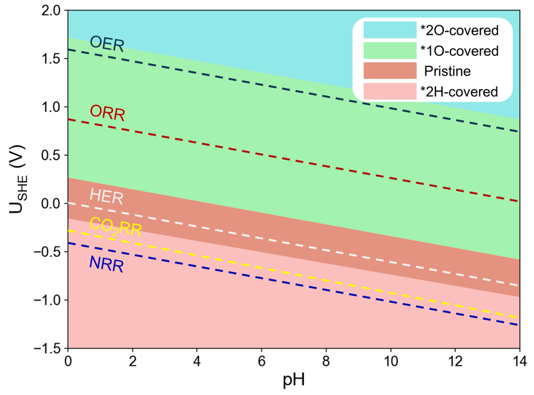

Fe-Ni-N$_{1,5,6}$-C

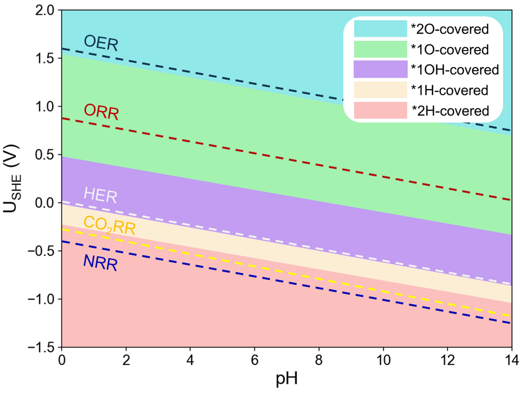

Fe-Ni-$N_{2,3,4}$-C	Fe-Ni-$N_{2,3,5}$-C

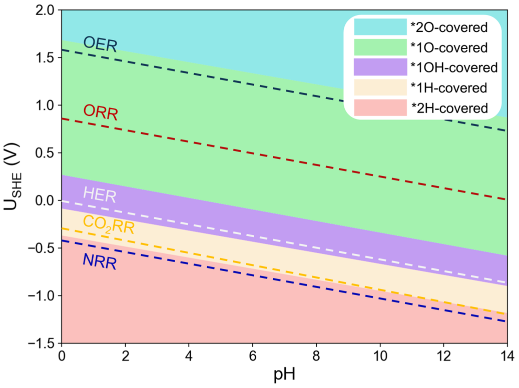

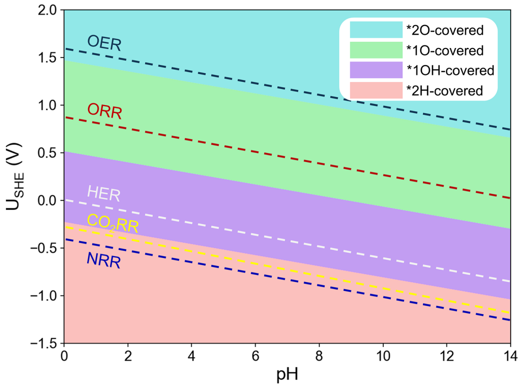

Fe-Ni-$N_{2,3,6}$-C	Fe-Ni-$N_{2,4,5}$-C

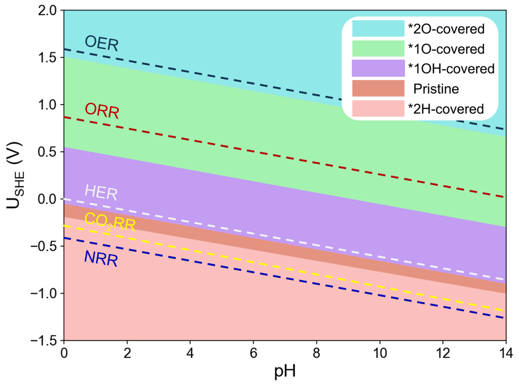

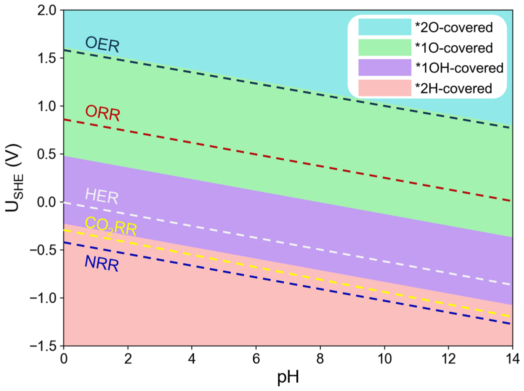

Fe-Ni-$N_{2,4,6}$-C	Fe-Ni-$N_{2,5,6}$-C

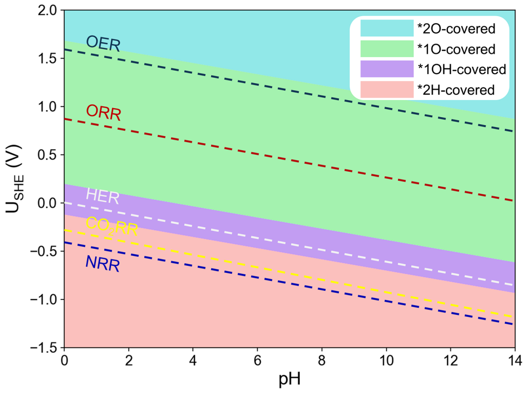

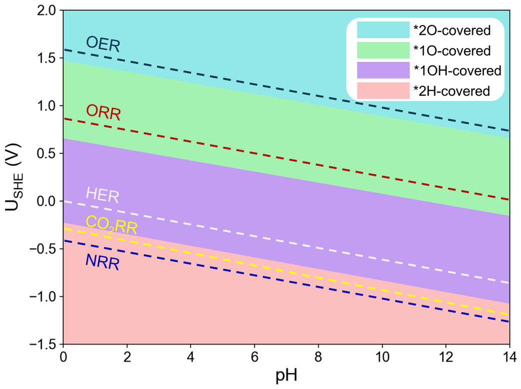

Fe-Ni-$N_{3,4,5}$-C	Fe-Ni-$N_{3,4,6}$-C

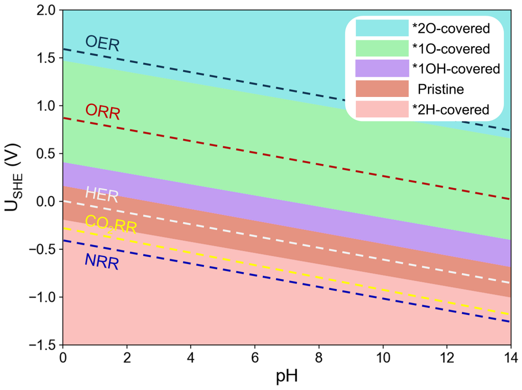

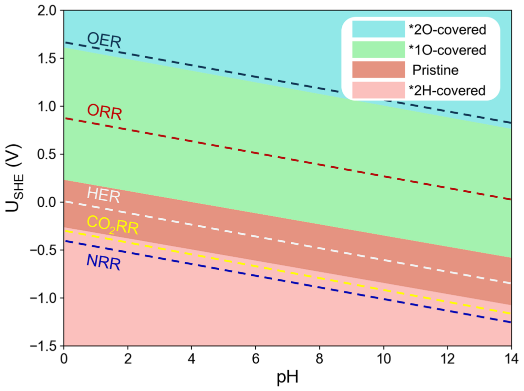

Fe-Ni-$N_{3,5,6}$-C

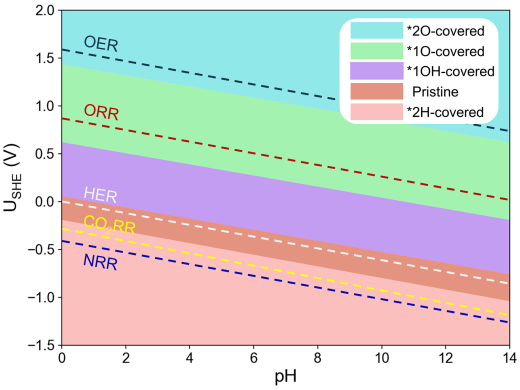

Fe-Ni-$N_{4,5,6}$-C

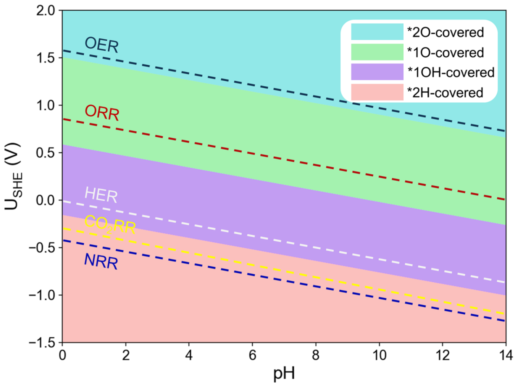

Fe-Ni-$N_{1,2,3,4}$-C

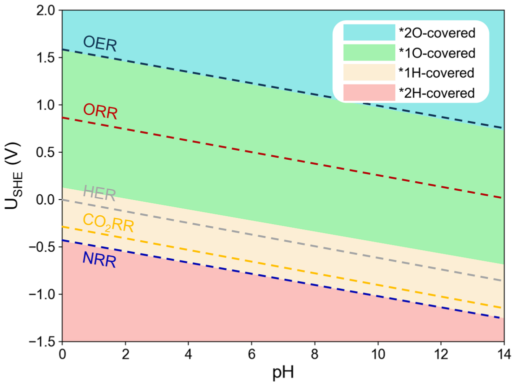

Fe-Ni-$N_{1,2,3,5}$-C

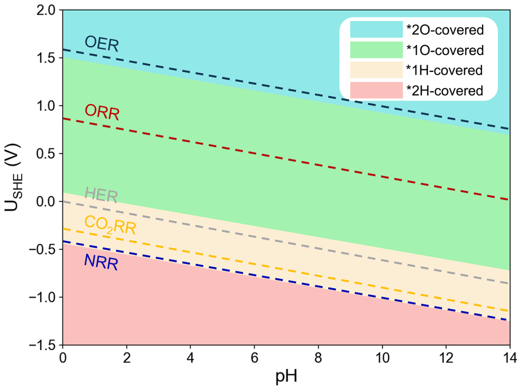

Fe-Ni-$N_{1,2,3,6}$-C

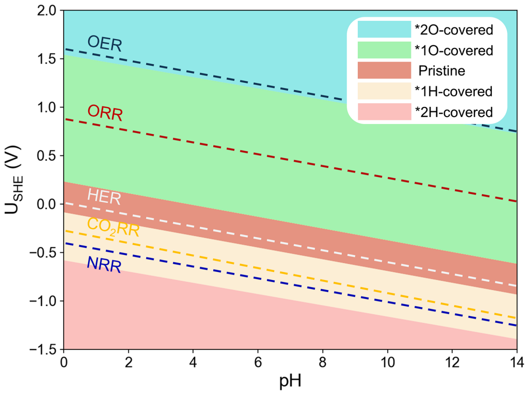

Fe-Ni-$N_{1,2,4,5}$-C

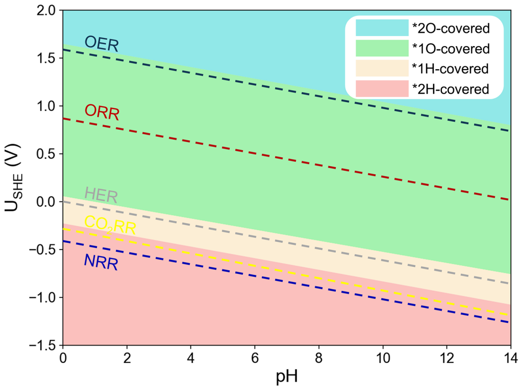

Fe-Ni-$N_{1,2,4,6}$-C

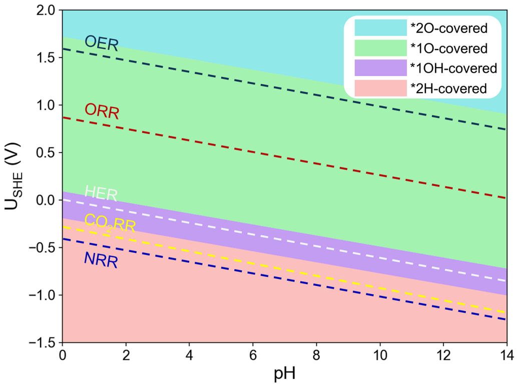

Fe-Ni-$N_{1,2,5,6}$-C

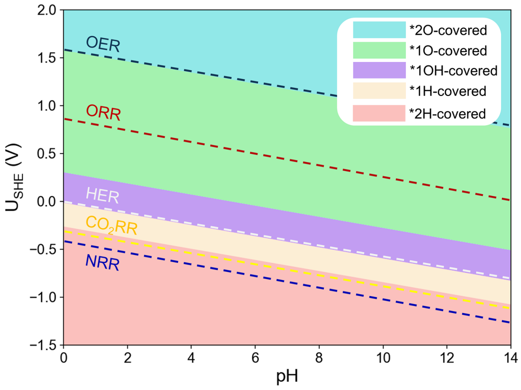

Fe-Ni-$N_{1,3,4,5}$-C	Fe-Ni-$N_{1,3,4,6}$-C

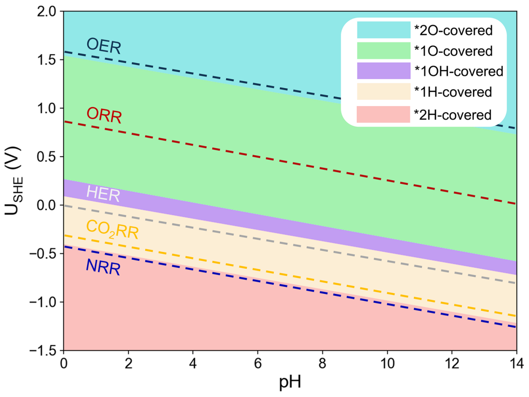

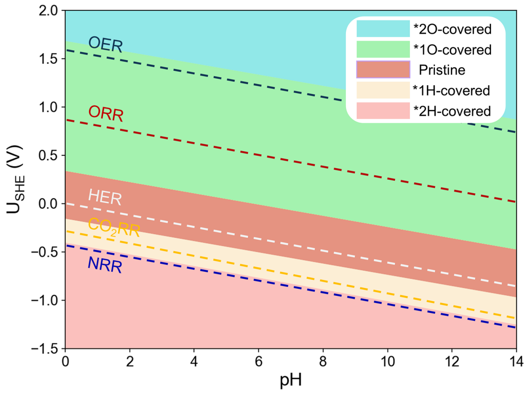

Fe-Ni-$N_{1,3,5,6}$-C	Fe-Ni-$N_{1,4,5,6}$-C

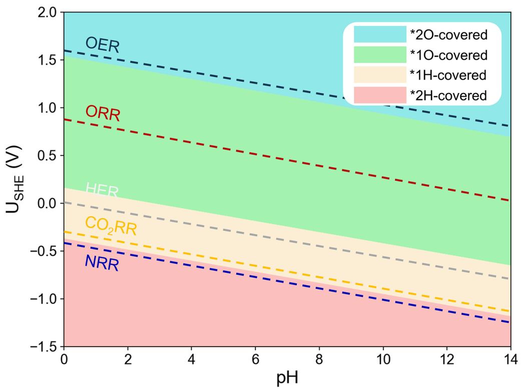

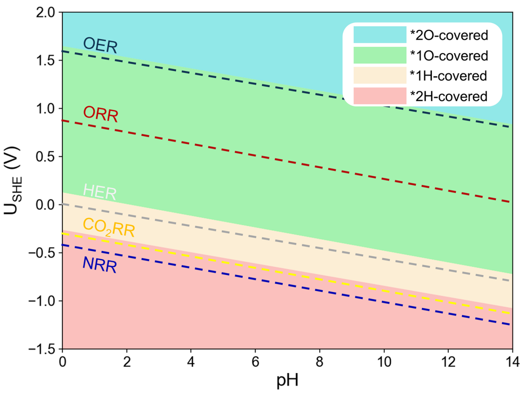

Fe-Ni-$N_{2,3,4,5}$-C	Fe-Ni-$N_{2,3,4,6}$-C

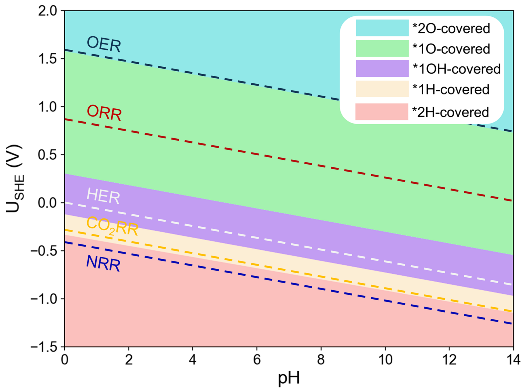

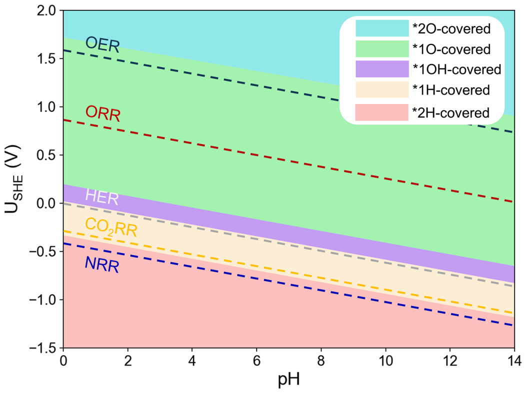

Fe-Ni-$N_{2,3,5,6}$-C	Fe-Ni-$N_{2,4,5,6}$-C

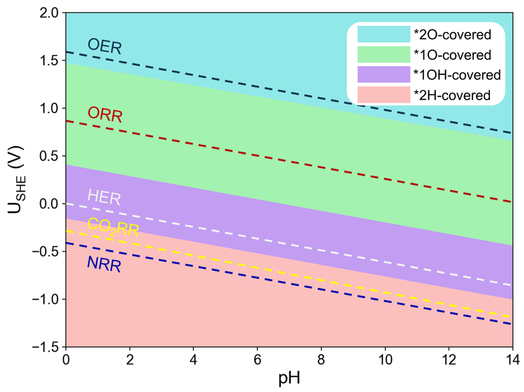

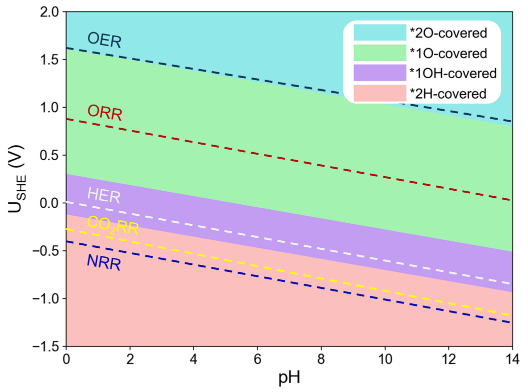

Fe-Ni-$N_{3,4,5,6}$-C

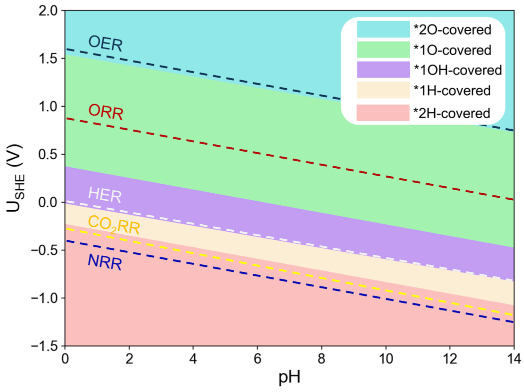

Fe-Ni-$N_{1,2,3,4,5}$-C

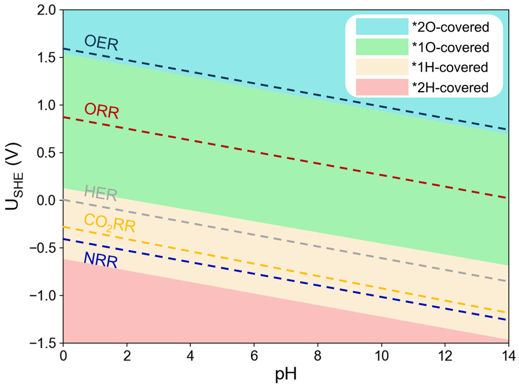

Fe-Ni-$N_{1,2,3,4,6}$-C

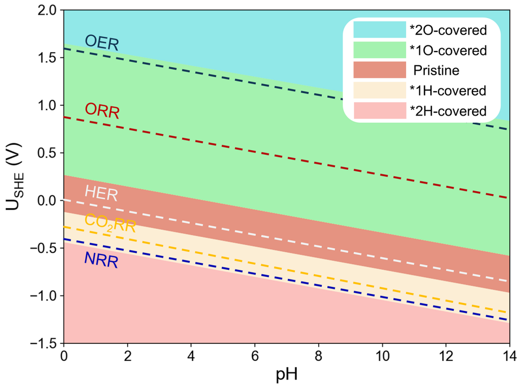

Fe-Ni-$N_{1,2,3,5,6}$-C

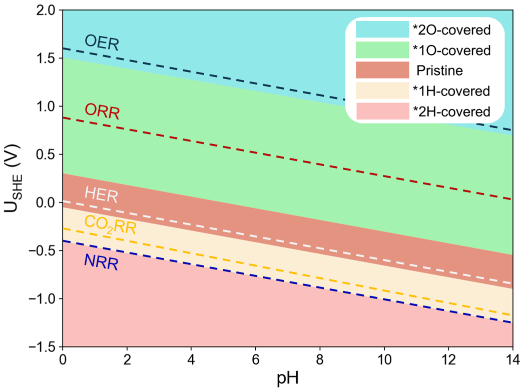

Fe-Ni-$N_{1,2,4,5,6}$-C

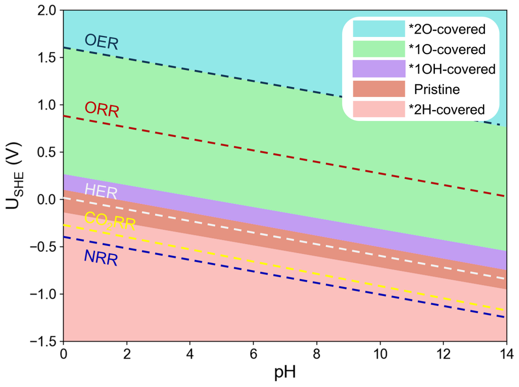

Fe-Ni-$N_{1,3,4,5,6}$-C

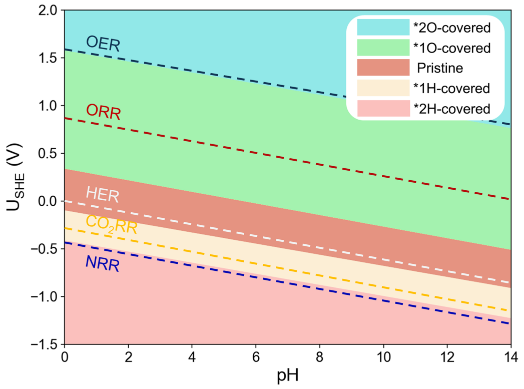

Fe-Ni-$N_{2,3,4,5,6}$-C

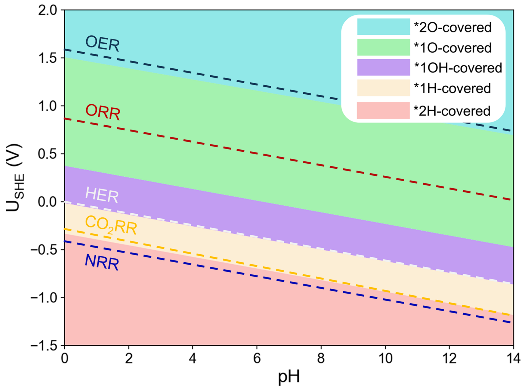

Fe-Ni-$N_{1,2,3,4,5,6}$-C

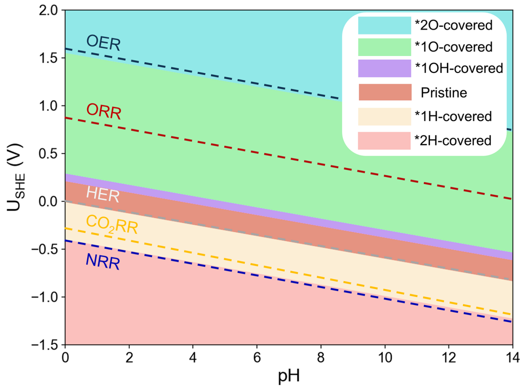

Supplementary Table 4. Surface states of Fe-Ni-N_x-C at the common potentials of HER, OER, ORR, CO_2RR, and NRR.

Models	Potential window (V)	HER (0 V)	OER (1.60 V)	ORR (0.78 V)	CO_2RR (-0.35 V)	NRR (-0.40V)
Fe-Ni-C	-0.36 ~ 0.28	Pristine	2O*	1O*	Pristine	2H*
Fe-Ni-N_{1}-C	-0.25 ~ 0.30	Pristine	2O*	1O*	2H*	2H*
Fe-Ni-N_{2}-C	-0.16 ~ 0.07	Pristine	2O*	2O*	2H*	2H*
Fe-Ni-N_{3}-C	-0.19 ~ 0.44	Pristine	2O*	1O*	2H*	2H*
Fe-Ni-N_{4}-C	-0.25 ~ 0.22	Pristine	2O*	1O*	2H*	2H*
Fe-Ni-N_{5}-C	-0.23 ~ 0.26	Pristine	2O*	1O*	2H*	2H*
Fe-Ni-N_{6}-C	-0.37 ~ 0.50	Pristine	2O*	1O*	Pristine	2H*
Fe-Ni-$N_{1,2}$-C	N/A	1HO*	2O*	1O*	2H*	2H*
Fe-Ni-$N_{1,3}$-C	-0.19 ~ 0.40	Pristine	2O*	1O*	2H*	2H*
Fe-Ni-$N_{1,4}$-C	-0.24 ~ 0.28	Pristine	1O*	1O*	2H*	2H*
Fe-Ni-$N_{1,5}$-C	N/A	1HO*	2O*	1O*	1H*	2H*
Fe-Ni-$N_{1,6}$-C	-0.25 ~ 0.37	Pristine	1O*	1O*	2H*	2H*
Fe-Ni-$N_{2,3}$-C	-0.19 ~ -0.05	1HO*	2O*	1O*	2H*	2H*
Fe-Ni-$N_{2,4}$-C	N/A	1HO*	1O*	1O*	2H*	2H*
Fe-Ni-$N_{2,5}$-C	N/A	1HO*	2O*	1OH*	1H*	1H*
Fe-Ni-$N_{2,6}$-C	N/A	1HO*	2O*	1OH*	2H*	2H*
Fe-Ni-$N_{3,4}$-C	-0.10 ~ 0.15	Pristine	1O*	1O*	2H*	2H*
Fe-Ni-$N_{3,5}$-C	-0.29 ~ 0.12	Pristine	2O*	1O*	2H*	2H*
Fe-Ni-$N_{3,6}$-C	-0.35 ~ 0.39	Pristine	2O*	1O*	Pristine	2H*
Fe-Ni-$N_{4,5}$-C	-0.24 ~ 0.02	Pristine	2O*	1O*	2H*	2H*
Fe-Ni-$N_{4,6}$-C	-0.29 ~ 0.25	Pristine	1O*	1O*	2H*	2H*
Fe-Ni-$N_{5,6}$-C	-0.19 ~ -0.03	1HO*	2O*	1O*	2H*	2H*
Fe-Ni-$N_{1,2,3}$-C	N/A	1HO*	2O*	1O*	1H*	1H*
Fe-Ni-$N_{1,2,4}$-C	N/A	1O*	1O*	1O*	1H*	2H*
Fe-Ni-$N_{1,2,5}$-C	N/A	1HO*	2O*	1O*	2H*	2H*
Fe-Ni-$N_{1,2,6}$-C	-0.16 ~ 0	Pristine	1O*	1O*	2H*	2H*
Fe-Ni-$N_{1,3,4}$-C	-0.16 ~ 0.33	Pristine	1O*	1O*	1H*	1H*

Fe-Ni-$N_{1,3,5}$-C	-0.08 ~ 0.13	Pristine	2O*	1O*	1H*	2H*
Fe-Ni-$N_{1,3,6}$-C	-0.08 ~ 0.22	Pristine	2O*	1O*	1H*	2H*
Fe-Ni-$N_{1,4,5}$-C	-0.08 ~ 0.18	Pristine	2O*	1O*	2H*	2H*
Fe-Ni-$N_{1,4,6}$-C	-0.17 ~ 0.28	Pristine	1O*	1O*	2H*	2H*
Fe-Ni-$N_{1,5,6}$-C	N/A	1HO*	2O*	1O*	2H*	2H*
Fe-Ni-$N_{2,3,4}$-C	N/A	1HO*	2O*	1O*	1H*	2H*
Fe-Ni-$N_{2,3,5}$-C	N/A	1HO*	2O*	1O*	2H*	2H*
Fe-Ni-$N_{2,3,6}$-C	-0.19 ~ -0.04	1HO*	2O*	1O*	2H*	2H*
Fe-Ni-$N_{2,4,5}$-C	N/A	1HO*	1O*	1O*	2H*	2H*
Fe-Ni-$N_{2,4,6}$-C	N/A	1HO*	1O*	1O*	2H*	2H*
Fe-Ni-$N_{2,5,6}$-C	N/A	1HO*	2O*	1O*	2H*	2H*
Fe-Ni-$N_{3,4,5}$-C	-0.19 ~ 0.16	Pristine	2O*	1O*	2H*	2H*
Fe-Ni-$N_{3,4,6}$-C	-0.26 ~ 0.25	Pristine	2O*	1O*	2H*	2H*
Fe-Ni-$N_{3,5,6}$-C	-0.18 ~ 0.05	Pristine	2O*	1O*	2H*	2H*
Fe-Ni-$N_{4,5,6}$-C	N/A	1HO*	2O*	1O*	2H*	2H*
Fe-Ni-$N_{1,2,3,4}$-C	N/A	1H*	2O*	1O*	1H*	1H*
Fe-Ni-$N_{1,2,3,5}$-C	N/A	1H*	2O*	1O*	1H*	1H*
Fe-Ni-$N_{1,2,3,6}$-C	-0.07 ~ 0.23	Pristine	2O*	1O*	1H*	1H*
Fe-Ni-$N_{1,2,4,5}$-C	N/A	1H*	1O*	1O*	2H*	2H*
Fe-Ni-$N_{1,2,4,6}$-C	N/A	1HO*	1O*	1O*	2H*	2H*
Fe-Ni-$N_{1,2,5,6}$-C	N/A	1HO*	2O*	1O*	2H*	2H*
Fe-Ni-$N_{1,3,4,5}$-C	N/A	1H*	2O*	1O*	1H*	2H*
Fe-Ni-$N_{1,3,4,6}$-C	-0.17 ~ 0.33	Pristine	1O*	1O*	1H*	2H*
Fe-Ni-$N_{1,3,5,6}$-C	N/A	1H*	2O*	1O*	1H*	2H*
Fe-Ni-$N_{1,4,5,6}$-C	N/A	1H*	1O*	1O*	2H*	2H*
Fe-Ni-$N_{2,3,4,5}$-C	N/A	1HO*	2O*	1O*	1H*	2H*
Fe-Ni-$N_{2,3,4,6}$-C	N/A	1H*	1O*	1O*	1H*	2H*
Fe-Ni-$N_{2,3,5,6}$-C	N/A	1HO*	2O*	1O*	2H*	2H*
Fe-Ni-$N_{2,4,5,6}$-C	N/A	1HO*	2O*	1O*	2H*	2H*
Fe-Ni-$N_{3,4,5,6}$-C	N/A	1HO*	2O*	1O*	2H*	2H*
Fe-Ni-$N_{1,2,3,4,5}$-C	N/A	1H*	2O*	1O*	1H*	1H*

Fe-Ni-$N_{1,2,3,4,6}$-C	-0.12 ~ 0.28	Pristine	1O*	1O*	1H*	1H*
Fe-Ni-$N_{1,2,3,5,6}$-C	-0.04 ~ 0.17	Pristine	2O*	1O*	1H*	1H*
Fe-Ni-$N_{1,2,4,5,6}$-C	-0.14 ~ 0.10	Pristine	2O*	1O*	2H*	2H*
Fe-Ni-$N_{1,3,4,5,6}$-C	-0.10 ~ 0.35	Pristine	2O*	1O*	1H*	2H*
Fe-Ni-$N_{2,3,4,5,6}$-C	N/A	1HO*	2O*	1O*	1H*	2H*
Fe-Ni-$N_{1,2,3,4,5,6}$-C	-0.01 ~ 0.21	Pristine	2O*	1O*	1H*	2H*
